# Food Consumption and Metabolic Risks in Young University Students

**DOI:** 10.3390/ijerph19010449

**Published:** 2021-12-31

**Authors:** Sughey González-Torres, Napoleón González-Silva, Ángel Pérez-Reyes, Luis Miguel Anaya-Esparza, Sergio Sánchez-Enríquez, Patricia N. Vargas-Becerra, Zuamí Villagrán, Maritza R. García-García

**Affiliations:** 1 Division de Ciencias Biomedicas, Centro Universitario de los Altos, Universidad de Guadalajara, Rafael Casillas Aceves 1200, Tepatitlan de Morelos 47620, Mexico; sgonzalez@cualtos.udg.mx (S.G.-T.); angel.preyes@academicos.udg.mx (Á.P.-R.); sergio.enriquez@cualtos.udg.mx (S.S.-E.); pvargas@cualtos.udg.mx (P.N.V.-B.); 2 Division de Ciencias Agropecuarias e Ingenierias, Centro Universitario de los Altos, Universidad de Guadalajara, Rafael Casillas Aceves 1200, Tepatitlan de Morelos 47620, Mexico; napoleon.gonzalez@cualtos.udg.mx (N.G.-S.); luis.aesparza@academicos.udg.mx (L.M.A.-E.); 3 Programa Internacional de Medicina, Unidad Academica de Ciencias de la Salud, Escuela de Medicina, Universidad Autónoma de Guadalajara, Lomas del Valle, Zapopan 45129, Mexico

**Keywords:** macronutrients, micronutrients, cytokines, adiponectin, interleukin-6, obesity

## Abstract

The purpose of this study was to analyze the association between components of the diet, metabolic risks, and the serum concentrations of adiponectin and interleukin-6 (IL-6). With prior informed consent, an analytical cross-sectional study was carried out with 72 students in their first year of university. The subjects had a mean age of 19.2 ± 1.0 years and body mass index of 23.38 ± 4.2, and they were mainly women (80.6%). Sociodemographic, anthropometric, and dietary data and metabolic risk factors were evaluated, and biochemical parameters and adipocytokines were also considered. The data were analyzed using means, ranges, and correlations, as well as principal components. In general, the protein, fat, and sodium intake were higher than the international dietary recommendations, and deficiencies in vitamins B5 and E, potassium, phosphorus, selenium, and zinc were observed. The most frequently observed metabolic risks were insulin resistance and hypoalphalipoproteinemia. IL-6 was positively correlated with lipid and protein intake. Adiponectin showed a positive correlation with high-density lipoprotein and a negative correlation with insulin, weight, and waist, while the adiponectin pattern was similar to that of vitamins E and A, which decreased with increasing intake of calories, macronutrients, and sodium. In general, a hypercaloric diet that was high in protein, fat, and sodium and deficient in vitamins, mainly fat-soluble, was associated with a lower concentration of adiponectin and a higher concentration of IL-6, which favor the presence of metabolic risks, including insulin resistance. Intervention studies are required to evaluate the dietary intake of metabolic markers in young people without comorbidities, which will lay the foundation for implementing prevention strategies.

## 1. Introduction

The prevalence and health impacts of obesity-related diseases are on the rise, doubling in more than 70 countries between the years of 1980 and 2015, and global estimates indicate that 604 million adults are obese [1]. Obesity is associated with immune responses that lead to a systemic low-grade proinflammatory state derived from an increase in adipocytes, loss of balance with immune cells, and the release of cytokines such as interleukin 6 (IL-6), which decreases adiponectin secretion [2,3]. In obesity, the IL-6 concentration increases, which decreases the sensitivity to insulin and increases glucose absorption in peripheral tissues [4,5,6], causing metabolic and cardiovascular diseases [7] and diabetes mellitus 2 [8,9]. Furthermore, adiponectin has been reported to increase adipocyte differentiation and inhibit IL-6 gene expression, which favors insulin sensitivity, preventing metabolic complications [10,11].

Obesity can be classified according to the distribution of body fat. Fat accumulated in the upper segment, associated with visceral fat deposits, decreases serum adiponectin levels [12,13,14]. Adiponectin is an adipokine with anti-inflammatory effects because it improves insulin sensitivity, regulates glucose tolerance, and increases fat metabolism [15,16]. Furthermore, it increases the secretion of interleukin 10 (IL-10), promoting the polarization of macrophages towards the M2 phenotype (anti-inflammatory mediator), and reduces the levels of tumor necrosis factor (TNFα) and IL-6 [17,18]. It should be noted that the adiponectin concentration depends on age, sex, weight, and diet.

Eating habits that include a high consumption of vegetables and fruits rich in antioxidants, cereals (preferably rich in dietary fiber), monounsaturated fats, and the frequent consumption of fish (compared to red meat or poultry) produce a higher adiponectin concentration, reducing the risk of metabolic alterations and consequent pathologies [19]. On the other hand, poor eating habits play a key role in body weight gain, which could lead to obesity and associated comorbidities [20]. In this context, young people attending university acquire inadequate lifestyle habits characterized by stress and working for many hours, promoting a greater consumption of processed products high in sugars and fats, coupled with a decrease in physical activity, which favors metabolic risks such as higher body mass index (BMI), an increase in the percentage of body fat, abdominal obesity, an increase in the waist-to-hip index, an increase in the waist-to-height index, insulin resistance, hyperglycemia, hypoalphalipoproteinemia, and hypertriglyceridemia, which lead to chronic non-communicable diseases [14,21,22,23]. Chronic inflammatory processes are usually associated with adulthood, particularly older adults (>70 years) with certain associated diseases [24,25,26]. However, the risk of developing inflammation begins during early life development [27]. Therefore, the objective of this study was to associate components of the diet with metabolic risks and serum concentrations of adiponectin and IL-6 in the young adult population.

## 2. Materials and Methods

### 2.1. Study Design and Participants

The present study was cross-sectional, and non-probabilistic convenience sampling was used to recruit first-year undergraduate students in the health area from a university in Los Altos de Jalisco, Mexico. Men and women aged between 18 and 22 years who had not been previously diagnosed with a chronic disease and were not consuming lipid-lowering drugs or weight loss medications were included. Pregnant or breastfeeding women were not considered in this study. Sociodemographic variables, including clinical history (hereditary family history and personal pathological history, including dependence on alcohol, tobacco, and other drugs), were collected based on the description of the medical history in the Mexican standard NOM-004-SSA3-2012 [28] to ensure that participants met the inclusion criteria. All participants provided their written consent, adhering to established ethical principles [29]. The project was approved by the Ethics and Research Committee of the Centro Universitario de los Altos of the University of Guadalajara (official letter no. CUA/CEI/DOBI005/2021).

### 2.2. Anthropometric and Dietary Measurement

Weight, height, and circumferences were evaluated with standardized procedures [30]. BMI and body fat percentage were obtained by bipolar bioelectric impedance using a Tbf-300A body analyzer (TANITA^®^, Tokyo, Japan). Dietary patterns were evaluated by means of 24 h recall (R24), which is a typical tabular tool that quantifies the food intake and beverages consumed throughout the day prior to the interview, describing the type of food (form of preparation, commercial brand, and food or vitamin supplements), the quantity, and the place and time of consumption. Evaluations were performed twice during the week and once on the weekend, from which the mean was calculated to estimate energy and macro- and micronutrient intake [31] using the Nutrikcal^®^VO software (Consinfo, Mexico City, Mexico).

### 2.3. Biochemical Assessment and Inflammation Markers

Blood samples were collected after fasting (>8 h) in vacutainer tubes (BD Vacutainer^®^, Stockholm, Sweden), which were centrifuged (Eppendorf^®^ 5810R, Hamburg, Germany) at 3260× *g*/15 min/4 °C to separate the serum. Biochemical determinations were analyzed in a Cobas C-111 instrument (Roche^®^, Basel, Switzerland) at the time of sample collection, while IL-6, adiponectin, and insulin were quantified by the sandwich ELISA technique using commercial kits (BioLegend, Inc., San Diego, CA, USA: IL-6 cat. no. 430501; adiponectin cat. no. 44304; and DRG^®^ International Inc., Springfield, NJ, USA: insulin cat. no. EIA-2935) following the manufacturer’s specifications. The absorbance measurements of the plates were obtained by using a microplate reader (Thermo Scientific™ Multiskan™ Go Microplate Spectrophotometer, Fisher Scientific S.L., Madrid, Spain) at 450 nm with the help of the CurveExpert software (Hyams Development, Chattanooga, TN, USA).

### 2.4. Clinical Evaluations and Metabolic Risk

Insulin resistance was assessed using the homeostatic model assessment for insulin resistance [HOMA-IR: Fasting insulin (µU/mL) × Fasting glucose (mmol/L)/22.5], where a HOMA-IR value > 2.5 was considered to be indicative of insulin resistance [32]. Values for hyperglycemia (>100 mg/dL), hypoalphalipoproteinemia (<40 mg/dL male; <50 mg/dL female), and hypertriglyceridemia (>150 mg/dL) were used [33]. In addition, excess body mass with BMI > 25 kg/m^2^ [34] and excess fat mass with a percentage of >33% in women and >20% in men [35] were considered risk values. Subsequently, the metabolic risk was considered on the basis of abdominal obesity (>85 cm women, >90 cm men) [36], waist–hip index (>0.85) [36], and waist–height index (>0.5) [37].

### 2.5. Statistical Analysis

Data are presented as mean ± standard deviation. For statistical analysis, normality tests were performed using the Kolmogorov–Smirnov test and Levene’s homogeneity of variance, analysis for comparison of means (Student’s t-test/one-way ANOVA, post hoc Tukey), sum of ranks (Mann–Whitney U/Kruskal–Wallis), chi-square or Fisher’s exact test, and Pearson’s correlation in SPSS v. 20 (SPSS^®^ Statics, IBM^®^, Chicago, IL, USA). Values of *p* < 0.05 were considered statistically significant. Principal component analysis (PCA) was performed to evaluate the behaviors of the variables and estimate their relationships among the study subjects. PCA was performed based on the means of triplicates of dietary patterns or single quantification of anthropometric and biochemical parameters, which were calculated without rotation, and the number of extracted factors was based on eigenvalues >1.0 and explained variance >70% using the Statistica software (v. 12 StatSoft^®^, Tulsa, OK, USA).

## 3. Results

### 3.1. Description of the Population and Diet Characterization

A total of 72 young university students in an age range of 18–22 years, mostly women (80.6%), who were enrolled in educational programs in health sciences, namely, nutrition (69.4%), medicine (18.1%), and nursing (12.5%), were evaluated. In general, only 26% of the population was overweight (higher prevalence in women, 17.2%) or obese (higher prevalence in men, 14.3%) (Table A1). Regarding food consumption, the distribution of macronutrients was as follows: carbohydrates (51%), proteins (17%), and fats (32%). In relation to micronutrients, deficiencies of vitamin A and magnesium were found in men, and calcium was deficient in women; deficiencies of vitamins B5 and E, potassium, phosphorus, selenium, and zinc, in addition to a high sodium intake, were found in both sexes (Table 1). However, no difference was found (*p* > 0.05) in the caloric intake between sexes (Table A1).

### 3.2. Food Consumption Associated with the Presence of Metabolic Risks

**Figure 1:**
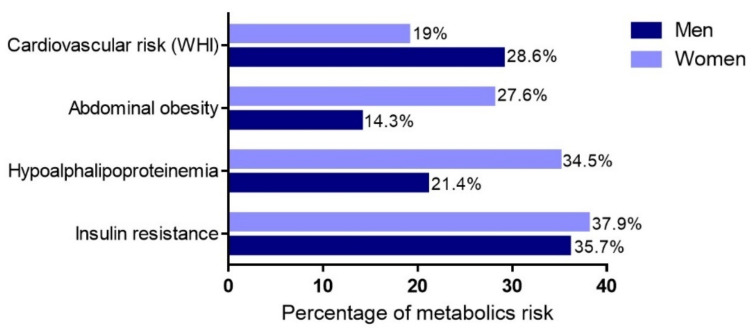
Metabolic risks by sex.

The metabolic risks in the population were insulin resistance (37.5%), hypoalphalipoproteinemia (31.9%), abdominal obesity (25%), and risk based on the waist–height index (20.8%). These risks did not significantly differ (*p* > 0.05) between sexes (Figure 1).

When contrasting the typical intake by food group, differences were found in legume consumption (*p* < 0.05) with respect to the insulin resistance condition and in fruit consumption (*p* < 0.01) with respect to the presence of hypoalphalipoproteinemia (Table 2 and Table 3). Likewise, in subjects with abdominal obesity, there was a trend towards greater consumption of legumes (Table 4), unlike those with cardiovascular risk, who did not show differences (*p* > 0.05) in the consumption of particular food groups (Table A2). In addition, the intake of macronutrients and micronutrients (Table A3, Table A4, Table A5 and Table A6) was associated with differences (*p* < 0.05) in the consumption of vitamin E in the presence of hypoalphalipoproteinemia and vitamins A and E in the presence of abdominal obesity (Table A4 and Table A5, respectively).

### 3.3. Dietary Consumption and Obesity in Relation to Adiponectin and IL-6 Concentrations

When evaluating the pattern of adiponectin in the presence of obesity and biochemical alterations, it was observed that greater weight, waist circumference, and concentrations of triglycerides, very low density lipoproteins, and insulin were associated with a decrease in the adiponectin concentration (*p* < 0.05) (Table 5). However, no differences were found (*p* > 0.05) in food consumption among adiponectin tertiles (Table A7).

On the other hand, the intake of vitamin B2 and selenium (Table 6) showed differences (*p* < 0.05) among IL-6 tertiles.

Likewise, the concentrations of glucose and cholesterol (Table 7) also showed differences (*p* < 0.05) among the tertiles of IL-6.

**Figure 2:**
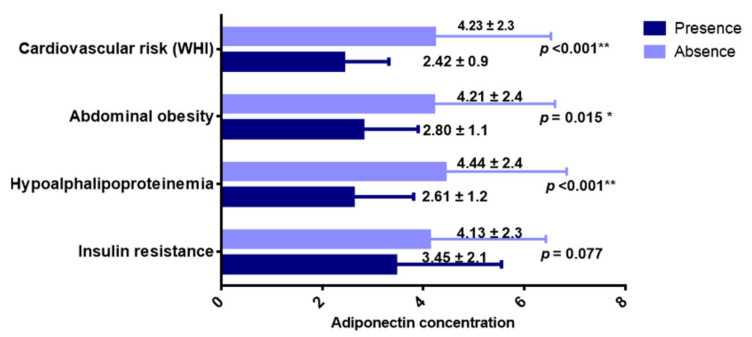
Adiponectin concentration (µg/mL) in the presence of metabolic risks. Data are shown as mean ± standard deviation. U Mann–Whitney. * *p* < 0.05. ** *p* < 0.01.

Regarding metabolic risks, adiponectin concentrations were lower in the presence of risk based on the waist–height index (*p* < 0.01), hypoalphalipoproteinemia (*p* < 0.01), and abdominal obesity (*p* < 0.05) (Figure 2). However, no differences (*p* > 0.05) were found to be associated with the IL-6 concentration (Table A8).

### 3.4. Principal Component Analysis among the Variables Analyzed

**Figure 3:**
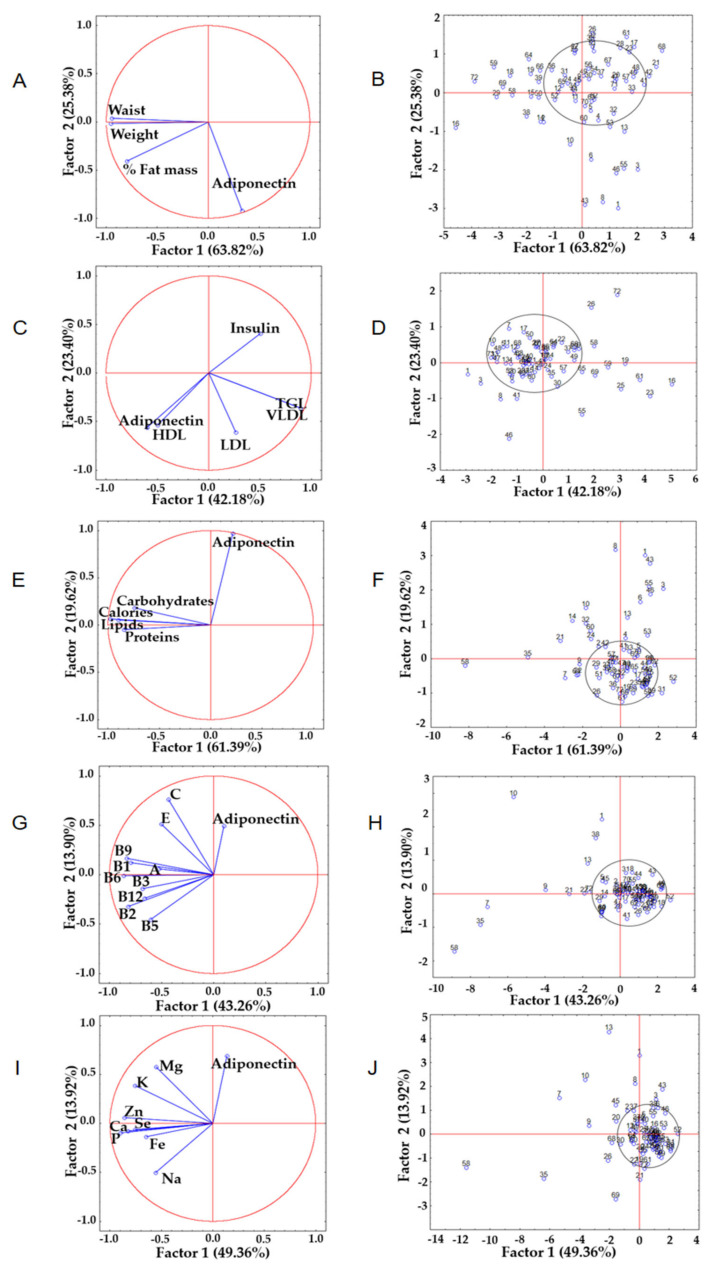
Principal component analysis (PCA). Left column: relationship between adiponectin and anthropometric parameters (**A**), biochemical parameters (**C**), caloric and macronutrient intake (**E**), vitamin intake (**G**), and mineral intake (**I**). Right column: distribution of pattern similarities among the studied subjects: anthropometric parameters (**B**), biochemical parameters (**D**), caloric and macronutrient intake (**F**), vitamin intake (**H**), and mineral intake (**J**).

Principal component analysis (PCA) is a statistical tool that considers a series of variables in a group of objects or individuals and, from them, calculates a new set of uncorrelated variables whose variances progressively decrease [39]. In this work, PCA was applied to identify associations between variables (adiponectin vs. anthropometric and biochemical parameters and dietary patterns) and determine patterns among study subjects, producing a unique factor solution (Figure 3). Regarding anthropometric parameters, principal component 1 (PC1) and principal component 2 (PC2) explained 89.2% of the total variance (PC1 63.8% and PC2 25.4%). In PC1, from positive to negative (Figure 3A), it was observed that with increasing adiponectin (0.33), the weight parameters (−0.95) and waist circumference (−0.94) decreased. This coincided with subjects who had a lower adiponectin concentration (quadrant II) and those who had a higher concentration (quadrant IV) (Figure 3B). On the other hand, the biochemical parameters showed a variance of 65.6% (PC1 42.2% and PC2 23.4%) (Figure 3C), which suggests that decreases in adiponectin values (−0.50) and high-density lipoproteins (−0.60) were accompanied by increases in the insulin concentration (0.51) and triglycerides (0.89) in PC1, reading from negative to positive. In addition, there was a separation by quadrants dependent on the values of insulin (quadrant I), adiponectin (quadrant III), and triglycerides (quadrant IV) in the study subjects (Figure 3D).

Regarding the dietary characteristics related to macronutrient intake, the PCA showed a variance of 81.0% (PC1 61.4% and PC2 19.6%) (Figure 3E), and it was observed that in PC2, from negative to positive, lower protein consumption (−0.05) was associated with an increase in the adiponectin concentration (0.97). In this sense, quadrant I grouped subjects with a higher concentration of adiponectin, and quadrant II contained subjects with a higher dietary intake, especially of proteins (Figure 3F). Additionally, in PC2 (total variance of 57.2%; PC1 43.3% and PC2 13.9%), it was observed that the consumption of vitamins C (0.76) and E (0.52) increased the concentration of adiponectin (0.49) (Figure 3G,H). Regarding mineral intake (Figure 3I), there was an increase in adiponectin concentrations (0.69) when sodium intake decreased (−0.50), as shown in PC2 (63.3% of the total variance, PC1 49.4% and PC2 13.9%) from positive to negative, while quadrant I included subjects with higher concentrations of adiponectin, and quadrant III clustered people with a high sodium intake (Figure 3J).

**Figure 4:**
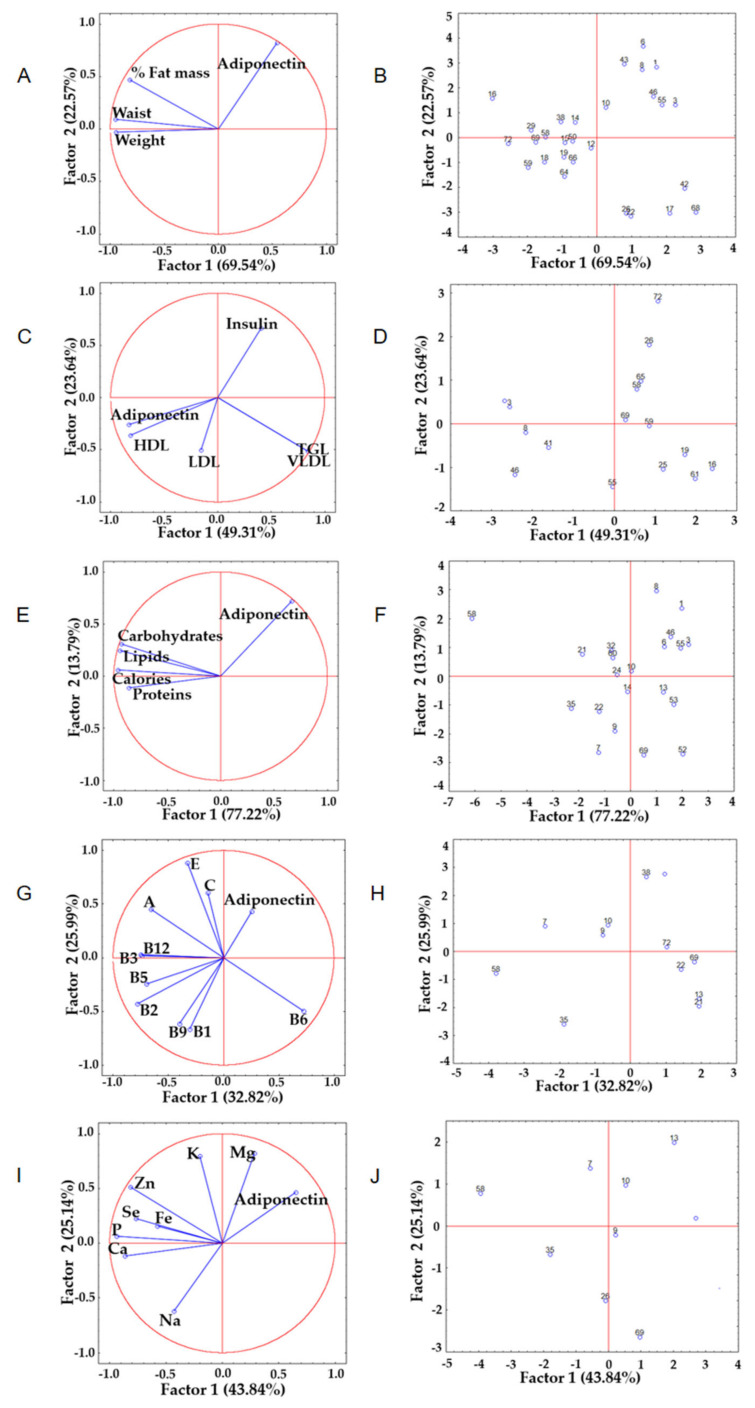
Principal component analysis (subgroup). Left column: relationship between adiponectin and anthropometric parameters (**A**), biochemical parameters (**C**), caloric and macronutrient intake (**E**), vitamin intake (**G**), and mineral intake (**I**). Right column: distribution of pattern similarities among subjects away from the initial set: anthropometric parameters (**B**), biochemical parameters (**D**), caloric and macronutrient intake (**F**), vitamin intake (**H**), and mineral intake (**J**).

Subsequently, a new PCA was performed for each of the parameters evaluated, in which only participants far from the initial set in the first analysis were considered (Figure 4). In general, a similar pattern of adiponectin among the total population was observed, with an increase in the percentages of variance (except for the PCA of vitamins). The anthropometric data (92.1% of the total variance; PC1 69.5% and PC2 22.6%) in PC1, from positive to negative, showed that adiponectin increased (0.54), while waist (−0.95), weight (−0.95), and fat percentage (−0.83) decreased (Figure 4A), consistent with observations in quadrants II and IV, which grouped the subjects with the highest and lowest fat percentages (Figure 4B). Biochemical indicators (variability of 72.9%; PC1 49.3% and PC2 23.6%) showed an increase in triglyceride values (0.84) and VLDL (0.84). There was also a reduction in the values of adiponectin (−0.83) and HDL (−0.82), as shown in PC1 from positive to negative (Figure 4C), consistent with observations in quadrant III, which grouped subjects with elevated HDL, and in quadrant IV, which grouped participants with higher triglyceride values (Figure 4D).

Additionally, when evaluating the intake of macronutrients (variance of 91.0%; PC1 77.2% and PC2 13.8%), the PC1 of the subgroup showed an increase in adiponectin (0.66) with the reduction in the consumption of calories (−0.96), carbohydrates (−0.93), lipids (−0.95), and protein (−0.86) (Figure 4E). From positive to negative, participants with higher levels of adiponectin were grouped in quadrant I, while quadrant IV clustered subjects who had the lowest intake of calories and carbohydrates (Figure 4F). In PC2, the intake of vitamins E (0.89), C (0.60), and A (0.45) promoted an increase in the adiponectin concentration (0.43) (variability of 58.8%; PC1 32.8% and PC2 26.0%) (Figure 4G), which coincided with subjects who had a higher intake of vitamin A (quadrant I) and vitamin E (quadrant II) (Figure 4H). Finally, the analysis of mineral intake revealed a variance of 68.9% (PC1 43.8% and PC2 25.1%), where it was observed that the values of adiponectin (0.46) and magnesium (0.82) were a function of sodium (−0.62) (PC2 from positive to negative, Figure 4I), while quadrant I grouped subjects with higher levels of adiponectin and magnesium intake, and quadrant III contained subjects with a high sodium intake (Figure 4J).

### 3.5. Relationship of the Anti-Inflammatory Cytokine Adiponectin and Inflammatory IL-6 with the Parameters Evaluated

**Figure 5:**
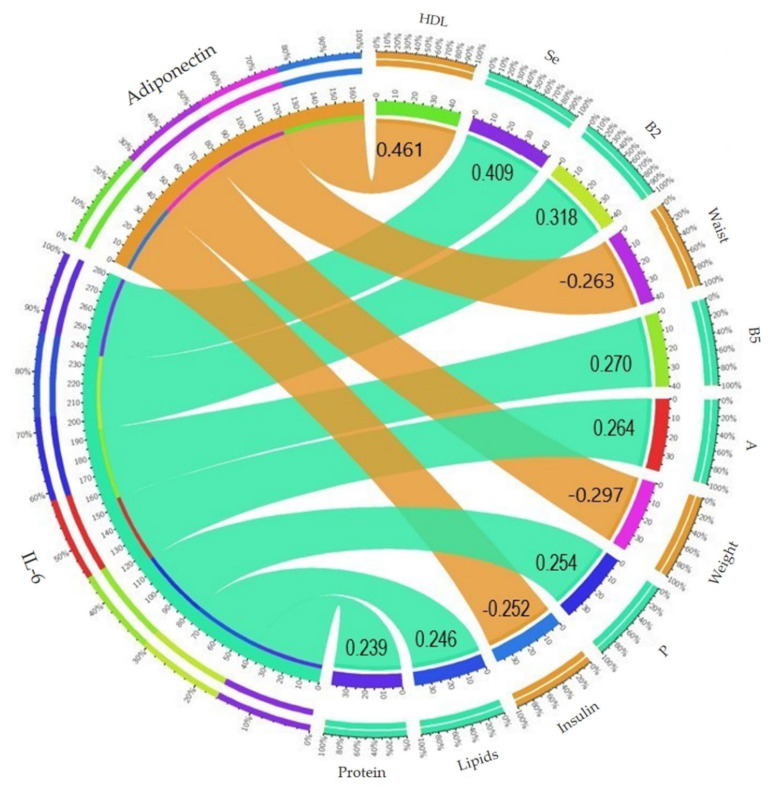
Correlation of adiponectin (orange shades) and interleukin-6 (green tones) with the variables evaluated (figure created in Circos Table Viewer software, vo. 63–9, Genome Sciences Centre, Vancouver, Canada).

In general, a positive correlation was observed between IL-6 and the consumption of lipids (0.246), proteins (0.239), vitamin A (0.264), vitamin B5 (0.270), phosphorus (0.254) (*p* < 0.05), vitamin B2 (0.318), and selenium (0.409) (*p* < 0.001), as well as between adiponectin and high-density lipoproteins (0.461) (*p* < 0.001). A negative correlation was identified between adiponectin and weight (−0.297), waist circumference (−0.263), and insulin levels (−0.252) (*p* < 0.05) (Figure 5).

## 4. Discussion

The most recent health and nutrition survey conducted in Mexico in 2020 reported a higher prevalence of obesity in women than in men for the population aged between 20 and 26 years [40]. These data are consistent with studies conducted in university populations of different Latin American countries [41,42]. Contrary to these reports, in the present study, it is men who showed higher rates of obesity; however, the accumulated percentages of overweight and obesity are similar to those reported by other authors [41,42].

In addition to the reported overweight and obesity, the percentage of underweight described in this study is above the percentage reported for Mexican youth by the National Health and Nutrition Survey [40]. For women, the values for obesity and underweight were equal. It should be noted that in the specific region where the work was carried out, the authors of a previous study among students with a similar age range (14–20 years) suggested that body dissatisfaction affected individuals at this particular stage, as young people seek sociocultural acceptance of their image [43], a condition that could affect food consumption. The authors found deficits in the intake of vitamin A (<900 mg/d), vitamin E (<15 mg/d), pantothenic acid B5 (<5.5 mg/d), and minerals such as potassium (<3500 mg/d), phosphorus (<1000 mg/d), selenium (<48 mg/d), and zinc (<12 mg/d) with respect to the recommended reference values for the Mexican population [38].

A deficiency in the intake of vitamins and minerals can have physiological implications because they function as cofactors of various enzymes. In addition, their deficiency favors oxidative stress, generating cellular damage in tissues and organs. Because of these micronutrient deficiencies (selenium and zinc), the glutathione peroxidase enzyme is inactive, and pro-oxidant enzymes are inhibited, which, together with α-tocopherol, prevent lipid peroxidation, increasing metabolic complications [44,45].

Vitamins A, C, and E are related to the concentration of adiponectin; a higher intake of these vitamins increases the concentration of adiponectin (an adipocytokine with anti-inflammatory, antiatherogenic, insulin-sensitizing, and antifibrotic effects) [15,16]. In general, the adiponectin concentration decreased in the population with metabolic risks, particularly those related to the waist-to-height index, hypoalphalipoproteinemia, and abdominal obesity, which is consistent with the literature [14,46,47]. This can be explained by the accumulation of visceral fat, mainly at the abdominal level, which increases the waist circumference and body weight with an associated decrease in adiponectin values, giving rise to high concentrations of triglycerides and very low density lipoproteins, since lipolysis in adipose tissue generates a supply of free fatty acids (FFAs) that go directly to the liver, producing triglycerides and VLDL. In addition, adipose tissue and skeletal muscle contain lipoprotein lipase, which can lead to hypertriglyceridemia [48].

On the other hand, regarding the consumption of macronutrients, high intake was found in the total percentage of fat (30%) and protein (0.8–1 g/kg/day), in addition to an increase in sodium intake (1 g/day), which exceeded the values established by international recommendations [33,49].

In health research, multivariate tools such as principal component analysis have been used to classify common patterns of consumption [50] or to categorize them by groups of metabolites evaluated [51]. The PCs showed lower concentrations of adiponectin with the high intake of proteins and sodium (general population) in addition to energy, macronutrients (especially carbohydrates), and sodium (subpopulation). It should be noted that young university students tend to increase their selection and consumption of processed products (characterized by high amounts of sugar, fat, and sodium) [22,52], which influence their body composition and generate metabolic alterations [23,53].

Adiponectin was positively correlated with HDL, which agrees with the literature [54,55] and emphasizes its anti-atherosclerotic and cardioprotective effects, consistent with its function [56,57]. Conversely, adiponectin was negatively correlated with insulin concentration. According to the literature, adiponectin promotes insulin and prevents insulin resistance by suppressing gluconeogenesis and inhibiting the expression of gluconeogenic enzymes (phosphoenol pyruvate carboxy-kinase and glucose 6-phosphatase); in addition, it suppresses the absorption of fatty acids and lipogenesis, while it increases the secretion of IL-10 by macrophages, promoting the polarization of macrophages towards the M2 phenotype, which is considered to be anti-inflammatory [17,18,48,58,59].

Subjects with low HDL values showed higher fruit consumption; however, this value could be due to the fact that only a subject in the group with the condition reported a high fruit intake (16.27 equivalents vs. 3.43 maximum range in the group without disease). It should be noted that even with said consumption, the subject was not overweight and did not have abdominal obesity, as reported in other studies [60,61]. It has been reported that an excess of simple sugars (glucose, fructose, and sucrose) contributes directly to an increase in lipogenesis, higher concentrations of LDL and VLDL, and therefore a decrease in circulating HDL [62]. Regarding the difference found in the consumption of vitamin E in association with both hypoalphalipoproteinemia and abdominal obesity, it is not considered relevant, because it does not comply with the recommended daily intake for the study population [38]. Despite this, the antioxidant activity of vitamins A and E (with statistical differences) could have reduced bioavailability because of meta-inflammation due to excess adiposity, especially abdominal [63].

In this study, the percentage of subjects with insulin resistance exceeds values reported in similar studies [41,42]. They showed that a higher consumption of legumes (8 equivalents/day maximum range), above the recommendation of 5 cups/week, provided beneficial effects for preventing obesity, diabetes, and metabolic syndrome, which were attributed to their high fiber content and nutrient density [64]. However, recent studies did not show that legumes had such effects [65,66], so further research is required.

The concentration of IL-6 showed a positive correlation with the consumption of lipids; proteins; vitamins A, B2, and B5; phosphorus; and selenium. A general explanation could be that IL-6 regulates various metabolic functions, including lipolysis, regulation of energy expenditure, and elimination of glucose when there is adequate signaling (canonical mode). However, in adiposity conditions, IL-6 (derived from adipocytes) generates different signaling (noncanonical mode) and affects the expected physiological regulation of metabolism [67]. In the specific case of vitamin A, a deficiency can affect immune functions related to inflammatory conditions [44,63]. On the other hand, selenium establishes a synergy with alpha-tocopherol for antioxidant defense [38,44]. A high-fat diet increases the IL-6 pathway, suggesting that a lower consumption of vegetable fat (olive oil, walnuts, almonds) coincides with vitamin and mineral deficiencies, as was reported previously. Thus, the consumption of foods considered to be proinflammatory that favor the secretion of IL-6 and other inflammatory cytokines should be assessed [68].

### Limitations

The limitations of this research may involve the observational study design, which does not allow the identification of causality, as well as the size and distribution of the sample, in which the female population had greater representation. However, historically, nutrition and nursing careers have comprised mostly women. In addition, it is important to consider that the students were studying health sciences, which may suggest a tendency towards health care; however, the selected students were in their first year of college, so their knowledge of health and nutrition would not necessarily be greater than that of the average population. Furthermore, obtaining information from a single educational space restricts the generalization of the findings to the population. To mitigate this issue, this research included university students from different municipalities of the state of Jalisco, Mexico, which was feasible because the evaluated institution is a regional university with a geographical location outside of the metropolitan area, receiving students from the entire area of Altos Sur de Jalisco.

On the other hand, the R24 has inherent limitations, but these limitations were mitigated during the research in the following aspects: it is known that responses to the R24 depend on the interviewee’s memory, so its application is not recommended in individuals who are elderly or younger than 12 years, but the age of our study population is not within these criteria. Likewise, a single R24 is not capable of estimating the habitual intake of an individual, so a total of three evaluations were performed on different days of the week, including weekends, and the results were averaged. It should be noted that the R24 was administered through face-to-face surveys by trained nutritionists, and possible biases in portion size and food ingredients were considered. Finally, the interview responses were evaluated using a database suitable for Mexican food composition.

## 5. Conclusions and Perspectives

In general, the eating and lifestyle habits of young people during university studies impact their adiponectin levels. In this context, one-third of the evaluated population of apparently healthy young undergraduate students presented insulin resistance or hypoalphalipoproteinemia with an increase in IL-6 and a decrease in adiponectin levels, which were associated with excessive dietary intake of calories, protein, sodium, and fat and deficiencies of mainly fat-soluble vitamins.

The identification of metabolic and cardiovascular risk factors at an early age allows the development of intervention programs or studies focused on determining, with greater precision, the influence of different diet components on metabolic alterations that lead to chronic diseases, particularly in the young popableulation without a previous diagnosis.

## Figures and Tables

**Table 1:** Intake of micronutrients according to the recommended daily intake (RDI).

Indicators	Total Intake (*n* = 72)	Men (*n* = 14)	Women (*n* = 58)	RDI
Vitamin A (mcg)	984.1 ± 925.9	820.3 ± 811.2 ^a^	1023.6 ± 953.7	>900
Vitamin B1 (mg)	1.4 ± 0.7	1.5 ± 1.0	1.3 ± 0.6	>1 M y >0.9 W
Vitamin B2 (mg)	1.6 ± 1.3	1.8 ± 1.5	1.6 ± 1.3	>1 M y >0.9 W
Vitamin B3 (mg)	17.8 ± 10.1	22.0 ± 16.9	16.8 ± 7.5	>13 M y >12 W
Vitamin B5 (mg)	3.0 ± 3.0	2.7 ± 2.4 ^a^	3.1 ± 3.2 ^a^	>5.5
Vitamin B6 (mg)	1.5 ± 0.9	1.7 ± 1.3	1.5 ± 0.8	>1.1
Vitamin B9 (mcg)	265.2 ± 195.7	254.2 ± 226.3	267.8 ± 189.8	>190
Vitamin B12 (mcg)	4.0 ± 4.6	5.1 ± 6.3	3.8 ± 4.1	>2.5
Vitamin C (mg)	110.5 ± 154.1	99.8 ± 68.0	113.0 ± 168.9	>84 M y >70 W
Vitamin E (mg)	2.1 ± 1.6	1.7 ± 1.6 ^a^	2.2 ± 1.6 ^a^	>15
Sodium (g)	2.5 ± 1.8	2.7 ± 1.6 ^b^	2.4 ± 1.9 ^b^	<1.6
Calcium (mg)	906.5 ± 486.9	1055.5 ± 696.9	870.5 ± 421.7 ^a^	>1000
Potassium (mg)	2204.3 ± 1098.0	2563.9 ± 1505.1 ^a^	2117.5 ± 927.4 ^a^	>3500
Magnesium (mg)	253.9 ± 134.2	238.1 ± 103.9 ^a^	257.7 ± 141.0	>320 M and >250 W
Phosphorus (mg)	769.0 ± 521.8	810.6 ± 556.0 ^a^	759.0 ± 517.7 ^a^	>1000
Selenium (mcg)	37.6 ± 51.8	31.6 ± 19.1 ^a^	39.0 ± 57.0 ^a^	>48
Zinc (mg)	7.4 ± 4.1	8.0 ± 5.4 ^a^	7.3 ± 3.8 ^a^	>12

Data are shown as mean ± standard deviation. The letters indicate ^a^ deficient intake and ^b^ excess intake. RDI: recommended daily intake for the Mexican population [38].

**Table 2:** Food consumption in the presence of insulin resistance.

Food Group	Total (*n* = 72)	Presence (*n* = 27)	Absence (*n* = 45)	*p*-Value
Fruit	1.59	1.70	1.54	0.253
Vegetables	2.79	2.12	3.18	0.722
Legumes	0.68	1.11	0.43	0.031
Cereals	8.71	9.19	8.45	0.193
Meats	6.09	5.76	6.23	0.142
Dairy	0.97	0.80	1.06	0.400
Fats	3.43	3.72	3.27	0.368
Sugars	3.20	2.58	3.42	0.674

Data presented as averages of equivalents. U Mann–Whitney. *p* < 0.05: statistically significant differences.

**Table 3:** Food consumption in the presence of hypoalphalipoproteinemia.

Food Group	Total (*n* = 72)	Presence (*n* = 23)	Absence (*n* = 49)	*p*-Value
Fruit	1.59	2.54	1.15	0.006
Vegetables	2.79	3.95	2.24	0.866
Legumes	0.68	0.67	0.68	0.229
Cereals	8.71	8.29	8.92	0.837
Meats	6.09	6.28	5.99	0.740
Dairy	0.97	0.93	0.99	0.758
Fats	3.43	3.30	3.50	0.933
Sugars	3.20	3.00	3.29	0.616

Data presented as averages of equivalents. U Mann–Whitney. *p* < 0.05: statistically significant differences.

**Table 4:** Food consumption in the presence of abdominal obesity.

Food Group	Total (*n* = 72)	Presence (*n* = 18)	Absence (*n* = 54)	*p*-Value
Fruit	1.59	1.84	1.51	0.128
Vegetables	2.79	4.73	2.13	0.198
Legumes	0.68	0.90	0.61	0.072
Cereals	8.71	8.40	8.82	0.998
Meats	6.09	6.79	5.85	0.979
Dairy	0.97	0.99	0.97	0.486
Fats	3.43	3.26	3.49	0.580
Sugars	3.20	2.70	3.36	0.820

Data presented as averages of equivalents. U Mann–Whitney. *p* < 0.05: statistically significant differences.

**Table 5:** Anthropometric and biochemical indicators with respect to adiponectin tertiles.

Indicators	Total Sample (100%, *n* = 72)	Tertile 1 <2.62 ng/dL (*n* = 24)	Tertile 2 >2.62–< 4.08 ng/dL (*n* = 24)	Tertile 3 >4.08 ng/dL (*n* = 24)	*p*-Value
Weight (kg) ^1^	63.3 ± 13.8	69.2 ± 15.5	60.4 ± 14.4	60.3 ± 11.8	0.037 ^a,b^
BMI (kg/m^2^) ^2^	23.4 ± 4.2	24.9 ± 4.8	22.4 ± 4.1	22.9 ± 3.4	0.206
Waist perimeter (cm) ^2^	75.0 ± 10.3	79.5 ± 9.3	73.1 ± 9.8	72.5 ± 10.6	0.009 ^a,c^
Body fat mass (%) ^1^	26.1 ± 9.8	26.8 ± 10.3	24.5 ± 11.0	26.9 ± 8.0	0.633
Glucose (mg/dL) ^1^	90.1 ± 5.9	90.8 ± 6.3	90.5 ± 6.5	89.0 ± 4.9	0.556
Cholesterol (mg/dL) ^1^	156.2 ± 27.2	151.6 ± 26.0	154.8 ± 22.2	162.2 ± 32.5	0.556
HDL-c (mg/dL) ^1^	55.7 ± 13.4	48.5 ± 10.3	56.1 ± 12.4	62.7 ± 13.8	0.001 ^a^
LDL-c (mg/dL) ^2^	85.5 ± 21.2	85.5 ± 23.3	85.2 ± 17.6	85.8 ± 23.2	0.956
VLDL-c (mg/dL) ^2^	14.9 ± 6.6	17.6 ± 7.2	13.5 ± 5.3	13.7 ± 6.6	0.026 ^a,c^
Triglycerides (mg/dL) ^2^	74.7 ± 33.1	88.0 ± 36.2	67.7 ± 26.6	68.4 ± 33.0	0.026 ^a,c^
Insulin (µUI/mL) ^2^	13.2 ± 8.9	17.6 ± 13.4	10.8 ± 3.2	11.3 ± 5.1	0.038 ^a,c^

Results are shown as mean ± standard deviation. ^1^ One-way ANOVA with a post hoc Tukey test. ^2^ Kruskal–Wallis test, post hoc Mann-Whitney. *p* < 0.05: statistically significant differences. ^a^ Tertile 1 vs. Tertile 3; ^b^ Tertile 2 vs. Tertile 3; ^c^ Tertile 1 vs. Tertile 2. BMI: body mass index; HDL-c: high-density cholesterol lipoprotein LDL-c: low-density cholesterol lipoprotein; VLDL-c: very low density cholesterol lipoprotein.

**Table 6:** Dietetic indicators with respect to IL-6 tertiles.

Indicators	Total Sample (*n* = 72)	Tertile 1 <1.53 pg/mL (*n* = 24)	Tertile 2 >1.53–<2.50 pg/mL (*n* = 24)	Tertile 3 >2.50 pg/mL (*n* = 24)	*p*-Value
Energy (kcal/day)	1939.4 ± 705.7	2068.1 ± 614.6	1933.4 ± 687.6	1816.8 ± 808.5	0.179
Carbohydrates (g)	243.3 ± 97.4	255.2 ± 82.9	266.3 ± 126.6	208.4 ± 66.2	0.131
Protein (g)	81.1 ± 35.7	86.1 ± 34.6	76.1 ± 23.8	81.1 ± 46.0	0.405
Lipids (g)	76.8 ± 37.8	80.0 ± 27.3	74.7 ± 32.0	75.7 ± 51.2	0.345
Vitamin A (mcg)	984.1 ± 925.9	975.9 ± 817.5	1109.1 ± 1057.3	885.2 ± 912.7	0.839
Vitamin B1 (mg)	1.4 ± 0.7	154 ± 0.8	1.4 ± 0.8	1.2 ± 0.5	0.406
Vitamin B2 (mg)	1.6 ± 1.3	1.8 ± 0.7	1.6 ± 1.1	1.6 ± 1.9	0.029 ^a,b^
Vitamin B3 (mg)	17.8 ± 10.1	19.2 ± 13.9	18.0 ± 9.5	16.2 ± 5.2	0.951
Vitamin B5 (mg)	3.0 ± 3.0	3.2 ± 2.3	2.6 ± 2.0	3.3 ± 4.4	0.123
Vitamin B6 (mg)	1.5 ± 0.9	1.8 ± 1.1	1.5 ± 0.9	1.3 ± 0.6	0.152
Vitamin B9 (mcg)	265.2 ± 195.7	321.3 ± 245.6	261.6 ± 165.6	212.7 ± 155.9	0.162
Vitamin B12 (mcg)	4.0 ± 4.6	5.1 ± 6.7	3.0 ± 2.2	4.0 ± 3.6	0.334
Vitamin C (mg)	110.5 ± 154.1	129.5 ± 226.1	100.7 ± 91.8	101.1 ± 115.1	0.830
Vitamin E (mg)	2.1 ± 1.6	2.2 ± 1.6	2.4 ± 1.7	1.8 ± 1.5	0.495
Sodium (g)	2.5 ± 1.8	2.3 ± 1.2	2.4 ± 1.4	2.7 ± 2.5	0.971
Calcium (mg)	906.5 ± 486.9	1014.2 ± 523.9	857.2 ± 386.1	848.1 ± 538.6	0.383
Potassium (mg)	2204.3 ± 1098.0	2558.0 ± 1494.4	1992.7 ± 644.2	2062.2 ± 935.4	0.420
Magnesium (mg)	253.9 ± 134.2	286.4 ± 162.4	235.6 ± 134.3	239.6 ± 97.6	0.413
Phosphorus (mg)	769.0 ± 521.8	853.4 ± 371.9	699.0 ± 374.0	754.6 ± 741.2	0.064
Selenium (mcg)	37.6 ± 51.8	41.8 ± 21.3	28.4 ± 14.3	42.5 ± 86.5	0.009 ^a,b^
Zinc (mg)	7.4 ± 4.1	8.3 ± 3.7	7.3 ± 3.9	6.7 ± 4.6	0.094

Data are shown as mean ± standard deviation. Kruskal-Wallis test, post hoc Mann-Whitney. ^a^ Tertile 1 vs. Tertile 3; ^b^ Tertile 1 vs. Tertile 2. *p* < 0.05: statistically significant differences.

**Table 7:** Anthropometric and biochemical indicators regarding the IL-6 tertiles.

Indicators	Total Sample (*n* = 72)	Tertile 1 <1.53 pg/mL (*n* = 24)	Tertile 2 >1.53–<2.50 pg/mL (*n* = 24)	Tertile 3 >2.50 pg/mL (*n* = 24)	*p*-Value
Weight (kg) ^1^	63.3 ± 13.8	62.3 ± 10.2	65.4 ± 16.2	62.2 ± 14.7	0.655
BMI (kg/m^2^) ^2^	23.4 ± 4.2	23.1 ± 3.2	23.6 ± 5.1	23.4 ± 4.3	0.957
Waist perimeter (cm) ^2^	75.0 ± 10.3	74.6 ± 8.8	76.8 ± 11.9	73.6 ± 10.0	0.468
Body fat mass (%) ^1^	26.1 ± 9.8	26.4 ± 10.0	25.9 ± 11.3	26.0 ± 8.1	0.983
Glucose (mg/dL) ^1^	90.1 ± 5.9	87.7 ± 4.7	90.1 ± 6.4	92.5 ± 5.8	0.017 ^a^
Cholesterol (mg/dL) ^1^	156.2 ± 27.2	150.3 ± 26.8	150.2 ± 22.4	168.0 ± 29.1	0.030 ^a,b^
HDL-c (mg/dL) ^1^	55.7 ± 13.4	54.9 ± 11.7	52.0 ± 11.3	60.3 ± 16.0	0.091
LDL-c (mg/dL) ^2^	85.5 ± 21.2	80.7 ± 22.5	82.7 ± 16.7	93.0 ± 22.6	0.173
VLDL-c (mg/dL) ^2^	14.9 ± 6.6	14.6 ± 7.8	15.5 ± 6.1	14.7 ± 6.1	0.499
Triglycerides (mg/dL) ^2^	74.7 ± 33.1	73.0 ± 38.8	77.6 ± 30.7	73.4 ± 30.4	0.499
Insulin (µUI/mL) ^2^	13.2 ± 8.9	12.3 ± 7.2	14.3 ± 12.3	13.1 ± 6.3	0.567

Data are shown as mean ± standard deviation. ^1^ One-way ANOVA with a post hoc Tukey test. ^2^ Kruskal-Wallis test, post hoc Mann-Whitney. ^a^ Tertile 1 vs. Tertile 3; ^b^ Tertile 2 vs. Tertile 3. *p* < 0.05: statistically significant differences. BMI: body mass index; HDL-c: high-density cholesterol lipoprotein; LDL-c: low-density cholesterol lipoprotein; VLDL-c: very low density cholesterol lipoprotein.

## Data Availability

The data presented in this study are available in the supplementary material.

## Supplementary Materials

The following are available online at https://www.mdpi.com/article/10.3390/ijerph19010449/s1.

## Appendix A

**Table A1:** General characteristics of the population evaluated regarding their sex.

Indicators	Total (*n* = 72)	Men (*n* = 14)	Women (*n* = 58)	*p*-Value
Sociodemographic				
^a^ Age (years)	19.2 ± 1.0	19.4 ± 1.3	19.1 ± 0.9	0.541
^b^ Tobacco (*n*)	19	4	15	0.539
^b^ Sedentary lifestyle (*n*)	37	6	31	0.340
Anthropometrics				
^c^ Weight (kg)	63.3 ± 13.8	70.8 ± 13.5	61.5 ± 13.4	0.032 *
^c^ Height (m)	1.64 ± 0.1	1.73 ± 0.1	1.62 ± 0.1	<0.001 **
^a^ Waist (cm)	75.0 ± 10.3	80.3 ± 8.5	73.7 ± 10.3	0.011 *
^c^ Fat mass (%)	26.1 ± 9.8	17.3 ± 8.3	28.2 ± 8.9	<0.001 **
^b^ Underweight (*n*)	6	1	5	0.670
^b^ Normal weight (*n*)	47	9	38	0.581
^b^ Overweight (*n*)	12	2	10	0.575
^b^ Obesity (*n*)	7	2	5	0.411
Dietetics				
^a^ Energy (kcal/day)	1939.4 ± 705.7	2199.1 ± 823.7	1876.8 ± 667.1	0.238
^a^ Carbohydrates (g/day)	243.3 ± 97.4	282.9 ± 112.4	233.8 ± 91.9	0.102
^a^ Protein (g/day)	81.1 ± 35.7	89.2 ± 46.2	79.1 ± 32.8	0.765
^a^ Lipids (g/day)	76.8 ± 37.8	84.8 ± 33.8	74.9 ± 38.7	0.238

Data are shown as mean ± standard deviation. ^a^ U Mann–Whitney; ^b^ chi-square or Fisher’s exact test; ^c^ Student’s t-test. * *p* < 0.05. ** *p* < 0.01. NS: no statistically significant differences.

**Table A2:** Food consumption in the presence of cardiovascular risk (W-H.I).

Food Group	Total (*n* = 72)	Presence (*n* = 15)	Absence (*n* = 57)	*p*-Value
Fruit	1.59	1.79	1.54	0.514
Vegetables	2.79	2.68	2.81	0.492
Legumes	0.68	0.77	0.66	0.141
Cereals	8.71	8.74	8.71	0.623
Meats	6.09	6.79	5.90	0.623
Dairy	0.97	1.01	0.96	0.505
Fats	3.43	2.4	3.40	0.657
Sugars	3.20	2.41	3.40	0.471

Data presented as averages of equivalents. U Mann–Whitney. *p* < 0.05: statistically significant differences. W-H.I: waist–height index.

**Table A3:** Dietary characteristics regarding insulin resistance.

Indicators	Total (*n* = 72)	Presence (*n* = 27)	Absence (*n* = 45)	*p*-Value
Energy (kcal/day)	1939.4 ± 705.7	2029.3 ± 825.0	1881.5 ± 633.9	0.636
Carbohydrates (g)	243.3 ± 97.4	246.9 ± 75.1	240.7 ± 110.5	0.303
Protein (g)	81.1 ± 35.7	82.2 ± 42.5	79.8 ± 31.6	0.981
Lipids (g)	76.8 ± 37.8	81.0 ± 50.0	74.4 ± 28.8	0.953
Vitamin A (mcg)	984.1 ± 925.9	1136.8 ± 1010.6	884.5 ± 878.5	0.207
Vitamin B1 (mg)	1.4 ± 0.7	1.5 ± 0.6	1.3 ± 0.8	0.140
Vitamin B2 (mg)	1.6 ± 1.3	1.8 ± 1.7	1.6 ± 1.0	0.957
Vitamin B3 (mg)	17.8 ± 10.1	16.2 ± 5.8	18.4 ± 11.9	0.850
Vitamin B5 (mg)	3.0 ± 3.0	2.9 ± 3.8	3.1 ± 2.6	0.390
Vitamin B6 (mg)	1.5 ± 0.9	1.5 ± 0.6	1.6 ± 1.0	0.976
Vitamin B9 (mcg)	265.2 ± 195.7	296.8 ± 207.6	246.2 ± 190.2	0.155
Vitamin B12 (mcg)	4.0 ± 4.6	4.4 ± 5.8	3.8 ± 3.8	0.308
Vitamin C (mg)	110.5 ± 154.1	108.7 ± 94.6	111.8 ± 183.9	0.164
Vitamin E (mg)	2.1 ± 1.6	2.4 ± 1.8	2.0 ± 1.5	0.328
Sodium (g)	2.5 ± 1.8	2.7 ± 2.4	2.3 ± 1.3	0.670
Calcium (mg)	906.5 ± 486.9	963.7 ± 535.4	864.3 ± 460.4	0.281
Potassium (mg)	2204.3 ± 1098.0	2303.4 ± 1084.7	2130.4 ± 1122.4	0.368
Magnesium (mg)	253.9 ± 134.2	281.2 ± 172.4	234.6 ± 103.0	0.488
Phosphorus (mg)	769.0 ± 521.8	834.7 ± 736.3	714.5 ± 327.2	0.749
Selenium (mcg)	37.6 ± 51.8	48.0 ± 81.7	31.1 ± 16.7	0.696
Zinc (mg)	7.4 ± 4.1	7.4 ± 4.7	7.4 ± 3.8	0.772

Data are shown as mean ± standard deviation. U Mann-Whitney. *p* < 0.05: statistically significant differences.

**Table A4:** Dietary characteristics regarding hypoalphalipoproteinemia.

Indicators	Total (*n* = 72)	Presence (*n* = 23)	Absence (*n* = 49)	*p*-Value
Energy (kcal/day)	1939.4 ± 705.7	1963.7 ± 863.2	1928.1 ± 628.2	0.677
Carbohydrates (g)	243.3 ± 97.4	240.8 ± 78.2	244.5 ± 105.9	0.823
Protein (g)	81.1 ± 35.7	82.6 ± 47.3	80.4 ± 29.2	0.731
Lipids (g)	76.8 ± 37.8	75.1 ± 50.5	77.59 ± 29.2	0.319
Vitamin A (mcg)	984.1 ± 925.9	1181.2 ± 1142.7	891.5 ± 801.4	0.305
Vitamin B1 (mg)	1.4 ± 0.7	1.4 ± 0.7	1.4 ± 0.7	0.633
Vitamin B2 (mg)	1.6 ± 1.3	1.9 ± 1.9	1.5 ± 0.9	0.562
Vitamin B3 (mg)	17.8 ± 10.1	16.1 ± 7.1	18.6 ± 11.3	0.608
Vitamin B5 (mg)	3.0 ± 3.0	3.4 ± 3.9	2.9 ± 2.6	0.133
Vitamin B6 (mg)	1.5 ± 0.9	1.6 ± 0.7	1.5 ± 0.9	0.196
Vitamin B9 (mcg)	265.2 ± 195.7	299.6 ± 218.9	249.0 ± 184.1	0.401
Vitamin B12 (mcg)	4.0 ± 4.6	4.7 ± 6.0	3.8 ± 3.8	0.909
Vitamin C (mg)	110.5 ± 154.1	157.0 ± 236.1	88.6 ± 89.9	0.169
Vitamin E (mg)	2.1 ± 1.6	2.8 ± 1.9	1.8 ± 1.4	0.013
Sodium (g)	2.5 ± 1.8	2.6 ± 2.6	2.4 ± 1.3	0.254
Calcium (mg)	906.5 ± 486.9	1005.1 ± 633.1	860.2 ± 400.2	0.616
Potassium (mg)	2204.3 ± 1098.0	2423.6 ± 1194.5	2101.4 ± 1046.7	0.274
Magnesium (mg)	253.9 ± 134.2	243.6 ± 124.7	258.7 ± 139.3	0.566
Phosphorus (mg)	769.0 ± 521.8	904.8 ± 771.2	705.2 ± 342.2	0.401
Selenium (mcg)	37.6 ± 51.8	50.6 ± 88.6	31.4 ± 15.7	0.904
Zinc (mg)	7.4 ± 4.1	7.5 ± 5.0	7.4 ± 3.6	0.510

Data are shown as mean ± standard deviation. U Mann–Whitney. *p* < 0.05: statistically significant differences.

**Table A5:** Dietary characteristics regarding abdominal obesity.

Indicators	Total (*n* = 72)	Presence (*n* = 18)	Absence (*n* = 54)	*p*-Value
Energy (kcal/day)	1939.4 ± 705.7	2018.9 ± 917.8	1913.0 ± 627.7	0.927
Carbohydrates (g)	243.3 ± 97.4	259.0 ± 114.7	238.1 ± 917.8	0.398
Protein (g)	81.1 ± 35.7	86.5 ± 51.7	79.3 ± 28.9	0.927
Lipids (g)	76.8 ± 37.8	79.6 ± 57.4	75.9 ± 29.2	0.558
Vitamin A (mcg)	984.1 ± 925.9	1435.8 ± 1240.4	833.5 ± 750.0	0.022
Vitamin B1 (mg)	1.4 ± 0.7	1.5 ± 0.6	1.3 ± 0.7	0.258
Vitamin B2 (mg)	1.6 ± 1.3	1.9 ± 2.1	1.6 ± 0.9	0.668
Vitamin B3 (mg)	17.8 ± 10.1	15.6 ± 6.1	18.5 ± 11.1	0.785
Vitamin B5 (mg)	3.0 ± 3.0	3.6 ± 4.5	2.8 ± 2.5	0.110
Vitamin B6 (mg)	1.5 ± 0.9	1.6 ± 0.6	1.5 ± 0.9	0.151
Vitamin B9 (mcg)	265.2 ± 195.7	288.2 ± 173.2	257.5 ± 203.6	0.180
Vitamin B12 (mcg)	4.0 ± 4.6	3.8 ± 3.8	4.1 ± 4.8	0.933
Vitamin C (mg)	110.5 ± 154.1	107.7 ± 103.9	111.4 ± 168.5	0.589
Vitamin E (mg)	2.1 ± 1.6	2.9 ± 1.8	1.9 ± 1.5	0.015
Sodium (g)	2.5 ± 1.8	2.8 ± 2.9	2.4 ± 1.3	0.668
Calcium (mg)	906.5 ± 486.9	951.5 ± 503.9	891.5 ± 485.1	0.413
Potassium (mg)	2204.3 ± 1098.0	2288.8 ± 900.8	2176.1 ± 1162.6	0.286
Magnesium (mg)	253.9 ± 134.2	257.8 ± 125.0	252.6 ± 138.2	0.682
Phosphorus (mg)	769.0 ± 521.8	862.8 ± 828.8	737.7 ± 374.6	0.917
Selenium (mcg)	37.6 ± 51.8	54.7 ± 98.5	31.9 ± 18.6	0.644
Zinc (mg)	7.4 ± 4.1	7.7 ± 5.5	7.4 ± 3.6	0.635

Data are shown as mean ± standard deviation. U Mann-Whitney. *p* < 0.05: no statistically significant differences.

**Table A6:** Dietary characteristics regarding the waist-height index.

Indicators	Total (*n* = 72)	Presence (*n* = 15)	Absence (*n* = 57)	*p*-Value
Energy (kcal/day)	1939.4 ± 705.7	2037.3 ± 984.8	1913.7 ± 621.0	0.884
Carbohydrates (g)	243.3 ± 97.4	234.9 ± 58.3	245.5 ± 105.6	0.745
Protein (g)	81.1 ± 35.7	85.6 ± 55.7	79.9 ± 28.8	0.718
Lipids (g)	76.8 ± 37.8	80.9 ± 61.1	75.7 ± 29.5	0.519
Vitamin A (mcg)	984.1 ± 925.9	1348.7 ± 1188.6	888.1 ± 829.9	0.095
Vitamin B1 (mg)	1.4 ± 0.7	1.4 ± 0.7	1.4 ± 0.7	0.862
Vitamin B2 (mg)	1.6 ± 1.3	1.9 ± 12.3	1.6 ± 0.9	0.693
Vitamin B3 (mg)	17.8 ± 10.1	15.9 ± 6.3	18.3 ± 10.9	0.683
Vitamin B5 (mg)	3.0 ± 3.0	3.6 ± 5.0	2.9 ± 2.4	0.983
Vitamin B6 (mg)	1.5 ± 0.9	1.5 ± 0.7	1.5 ± 0.9	0.698
Vitamin B9 (mcg)	265.2 ± 195.7	263.1 ± 199.7	265.7 ± 196.5	0.983
Vitamin B12 (mcg)	4.0 ± 4.6	4.0 ± 4.1	4.0 ± 4.8	0.906
Vitamin C (mg)	110.5 ± 154.1	106.0 ± 108.7	111.6 ± 164.8	0.835
Vitamin E (mg)	2.1 ± 1.6	2.6 ± 2.1	2.0 ± 1.4	0.454
Sodium (g)	2.5 ± 1.8	3.1 ± 3.1	2.3 ± 1.3	0.755
Calcium (mg)	906.5 ± 486.9	929.4 ± 562.5	900.5 ± 470.5	0.906
Potassium (mg)	2204.3 ± 1098.0	2216.5 ± 1005.5	2101.1 ± 1129.5	0.766
Magnesium (mg)	253.9 ± 134.2	249.5 ± 135.0	255.0 ± 135.1	0.890
Phosphorus (mg)	769.0 ± 521.8	878.8 ± 919.4	740.1 ± 360.2	0.808
Selenium (mcg)	37.6 ± 51.8	54.4 ± 108.6	33.2 ± 18.8	0.401
Zinc (mg)	7.4 ± 4.1	7.3 ± 5.7	7.5 ± 3.6	0.270

Data are shown as mean ± standard deviation. U Mann-Whitney. *p* < 0.05: statistically significant differences.

**Table A7:** Dietary characteristics regarding adiponectin concentrations.

Indicators	Total (100%, *n* = 72)	Tertile 1 <2.62 ng/dL (*n* = 24)	Tertile 2 >2.62–<4.08 ng/dL (*n* = 24)	Tertile 3 >4.08 ng/dL (*n* = 24)	*p*-Value
Energy (kcal/day)	1939.4 ± 705.7	2107.6 ± 938.0	1841.6 ± 566.4	1869.1 ± 540.2	0.806
Carbohydrates (g)	243.3 ± 97.4	258.2 ± 99.9	221.4 ± 61.8	250.4 ± 121.1	0.624
Protein (g)	81.1 ± 35.7	89.8 ± 55.3	078.7 ± 20.6	74.7 ± 18.0	0.683
Lipids (g)	76.8 ± 37.8	83.6 ± 51.6	71.2 ± 30.7	75.6 ± 26.8	0.690
Vitamin A (mcg)	984.1 ± 925.9	1110.9 ± 1138.4	904.7 ± 813.9	936.5 ± 813.8	0.912
Vitamin B1 (mg)	1.4 ± 0.7	1.5 ± 0.9	1.3 ± 0.5	1.2 ± 0.7	0.623
Vitamin B2 (mg)	1.6 ± 1.3	2.1 ± 2.1	1.4 ± 0.7	1.4 ± 0.5	0.544
Vitamin B3 (mg)	17.8 ± 10.1	20.1 ± 13.8	17.8 ± 7.5	15.5 ± 7.6	0.257
Vitamin B5 (mg)	3.0 ± 3.1	3.5 ± 4.2	2.8 ± 2.3	2.8 ± 2.5	0.879
Vitamin B6 (mg)	1.5 ± 0.9	1.7 ± 1.1	1.5 ± 0.7	1.4 ± 0.8	0.457
Vitamin B9 (mcg)	265.2 ± 195.7	285.6 ± 220.2	247.8 ± 139.7	262.3 ± 222.5	0.870
Vitamin B12 (mcg)	4.0 ± 4.6	5.9 ± 7.3	3.0 ± 1.7	3.2 ± 2.0	0.486
Vitamin C (mg)	110.5 ± 154.2	109.2 ± 79.3	85.5 ± 85.4	136.7 ± 241.7	0.451
Vitamin E (mg)	2.1 ± 1.6	2.3 ± 1.7	2.0 ± 1.7	201 ± 1.5	0.775
Sodium (g)	2.5 ± 1.8	3.1 ± 2.6	2.3 ± 1.3	2.1 ± 1.8	0.446
Calcium (mg)	906.5 ± 486.9	1063.2 ± 676.3	779.8 ± 364.8	876.5 ± 313.2	0.237
Potassium (mg)	2204.3 ± 1098.0	2403.7 ± 1384.7	2030.4 ± 559.5	2178.8 ± 1190.6	0.863
Magnesium (mg)	253.8 ± 134.2	236.3 ± 112.5	283.5 ± 131.6	241.8 ± 155.6	0.248
Phosphorus (mg)	769.0 ± 521.8	931.6 ± 804.5	740.5 ± 342.7	634.8 ± 168.4	0.763
Selenium (mcg)	37.6 ± 51.8	47.5 ± 87.5	31.6 ± 14.6	33.7 ± 15.8	0.632
Zinc (mg)	7.4 ± 4.1	8.1 ± 6.0	7.0 ± 2.6	7.2 ± 2.8	0.637

Data are shown as mean ± standard deviation. Kruskal–Wallis test, post hoc Mann-Whitney. *p* < 0.05: statistically significant differences.

**Table A8:** IL-6 concentration in presence of metabolic risks.

Indicators	Presence	Absence	*p*-Value
Insulin resistance	2.83 ± 3.1 (*n* = 27)	2.34 ± 3.3 (*n* = 45)	0.403
hypoalphalipoproteinemia	2.21 ± 3.2 (*n* = 23)	2.67 ± 3.2 (*n* = 49)	0.208
Overweight and obesity	3.14 ± 3.6 (*n* = 19)	2.38 ± 3.1 (*n* = 47)	0.575
Abdominal obesity	3.10 ± 3.7 (*n* = 18)	2.30 ± 3.0 (*n* = 54)	0.527
Risk (waist–height)	3.58 ± 3.9 (*n* = 15)	2.24 ± 2.9 (*n* = 57)	0.291
Risk (waist–hip)	1.0 ± 1.3 (*n* = 2)	2.57 ± 3.2 (*n* = 70)	0.230

Data are shown as mean ± standard deviation. U Mann-Whitney. *p* < 0.05: statistically significant differences.
