# Supplementary material for: Food Consumption and Metabolic Risks in Young University Students
Source: Int J Environ Res Public Health. 2021 Dec 31;19(1):449. doi: 10.3390/ijerph19010449 (PMC8744619; doi:10.3390/ijerph19010449)
Supplement: Supplementary file 1 [file ijerph-19-00449-s001.zip › ijerph-1509286-supplementary.pdf]

Folio	Course	Gender	Age	Marital.status	Smokig	Weight	Height	BMI	Classif.BMI	Weight.excess
1	6	2	21	1	0	55.8	1.59	22.07	1	0
2	6	2	20	1	0	75.1	1.67	26.93	2	1
3	6	2	19	1	0	48.9	1.54	20.62	1	0
4	6	2	21	1	0	58.1	1.66	21.08	1	0
5	6	2	20	1	0	57.5	1.57	23.33	1	0
6	6	2	19	1	0	63.5	1.65	23.32	1	0
7	6	1	19	1	0	64.6	1.72	21.84	1	0
8	6	2	20	1	0	58.1	1.59	22.98	1	0
9	6	2	19	1	0	63.1	1.59	24.96	1	0
10	6	2	19	1	0	64.3	1.64	23.91	1	0
11	6	2	20	1	1	66.4	1.68	23.53	1	0
12	6	2	19	1	1	68.4	1.7	23.67	1	0
13	6	2	22	1	0	55.6	1.67	19.94	1	0
14	5	2	19	1	1	77.7	1.66	28.20	2	1
15	5	2	19	1	0	81.6	1.78	25.75	2	1
16	5	2	19	1	0	97.9	1.68	34.69	3	1
17	5	1	18	1	1	52	1.72	17.58	0	0
18	6	1	19	1	0	94.8	1.77	30.26	3	1
19	6	2	20	1	0	72.2	1.58	28.92	2	1
20	6	2	21	1	0	50.5	1.5	22.44	1	0
21	6	2	19	1	0	42.5	1.58	17.02	0	0
22	5	1	18	1	1	65.7	1.78	20.74	1	0
23	5	2	18	1	0	44.3	1.51	19.43	1	0
24	5	1	19	1	0	74.4	1.77	23.75	1	0
25	5	1	20	1	0	69	1.77	22.02	1	0
26	5	1	22	1	1	62.6	1.68	22.18	1	0
27	5	1	20	1	1	73.1	1.82	22.07	1	0
28	5	1	19	1	0	58	1.75	18.94	1	0
29	5	2	18	1	1	82.3	1.61	31.75	2	1
30	6	2	19	1	0	58.9	1.64	21.90	1	0
31	5	2	19	1	0	66.9	1.69	23.42	1	0
32	6	2	20	1	0	50.8	1.61	19.60	1	0
33	6	2	18	1	1	46.4	1.49	20.90	1	0
34	6	2	19	1	0	66	1.64	24.54	1	0
35	6	1	19	1	0	66.3	1.77	21.16	1	0
36	6	2	19	1	0	66.1	1.67	23.70	1	0
37	6	2	18	1	0	54.1	1.59	21.40	1	0
38	6	2	18	1	0	79.1	1.6	30.90	3	1
39	6	2	19	1	0	71.9	1.58	28.80	2	1
40	6	2	19	1	0	47.4	1.6	18.52	1	0
41	6	2	18	1	1	43.6	1.49	19.64	1	0
42	6	2	18	1	1	45.1	1.57	18.30	0	0
43	6	2	19	1	0	65.1	1.61	25.11	2	1
44	6	2	20	1	0	68	1.79	21.22	1	0
45	6	2	19	1	1	55.8	1.44	26.91	2	1
46	6	2	21	1	1	56.5	1.58	22.63	1	0

47	6	2	20	1	0	50.4	1.61	19.44	1	0
48	6	2	19	1	0	44.4	1.58	17.79	0	0
49	6	2	19	1	0	57.7	1.6	22.54	1	0
50	6	2	20	1	0	75.1	1.7	25.99	2	1
51	6	1	18	1	0	62.2	1.64	23.13	1	0
52	6	2	19	1	1	68.7	1.68	24.34	1	0
53	6	2	18	1	0	57.8	1.69	20.24	1	0
54	6	2	18	1	0	52.5	1.61	20.25	1	0
55	6	2	18	1	1	55.6	1.64	20.67	1	0
56	6	2	19	1	0	56.3	1.61	21.72	1	0
57	6	2	18	1	0	47.4	1.6	18.52	1	0
58	6	2	18	1	0	84.3	1.67	30.23	3	1
59	6	1	19	1	0	102	1.77	32.56	3	1
60	6	2	19	1	0	58.7	1.65	21.56	1	0
61	6	2	20	1	0	46.5	1.55	19.35	1	0
62	6	2	18	1	0	60.3	1.63	22.70	1	0
63	6	2	19	1	1	58.3	1.61	22.49	1	0
64	4	1	20	1	0	78.6	1.66	28.52	2	1
65	4	1	22	1	0	67.8	1.61	26.16	2	1
66	4	2	20	1	1	70.4	1.64	26.17	2	1
67	4	2	19	1	1	51.1	1.71	17.48	0	0
68	4	2	19	1	0	41.1	1.55	17.11	0	0
69	4	2	18	1	0	87	1.62	33.15	3	1
70	4	2	19	1	1	62.9	1.62	23.97	1	0
71	4	2	20	1	0	54.3	1.63	20.44	1	0
72	4	2	18	1	0	99.6	1.7	34.46	3	1

Waist	Abdominal.obesity	Hip	Waist-hip.Index	Waist-Hip.Risk	Waist-height.Index
67	0	90	0.7444	2	0.42138
81.2	1	113	0.7186	1	0.48623
63	0	90.5	0.6961	1	0.40909
68.7	0	101	0.6802	1	0.41386
74	0	92	0.8043	3	0.47134
73	0	100	0.73	2	0.44242
76	0	90	0.8444	3	0.44186
75	0	98	0.7653	2	0.4717
70	0	98	0.7143	1	0.44025
77.5	0	106.2	0.7298	1	0.47256
71	0	102	0.6961	1	0.42262
75	0	101	0.7426	2	0.44118
62	0	84	0.7381	2	0.37126
80.5	1	110	0.7318	2	0.48494
83	1	108	0.7685	2	0.46629
111.3	1	125.5	0.8869	3	0.6625
67	0	84	0.7976	2	0.38953
89.5	0	111	0.8063	3	0.50565
88.5	1	108	0.8194	3	0.56013
69	0	93	0.7419	2	0.46
60	0	86.5	0.6936	1	0.37975
76.5	0	98	0.7806	2	0.42978
67	0	88	0.7614	2	0.44371
85	0	98	0.8673	3	0.48023
84.5	0	97.5	0.8667	3	0.4774
73.5	0	93.5	0.7861	2	0.4375
80	0	100	0.8	3	0.43956
70	0	87	0.8046	3	0.4
98.5	1	115	0.8565	3	0.6118
71	0	96.5	0.7358	2	0.43293
75	0	105.5	0.7109	1	0.44379
70	0	92	0.7609	2	0.43478
63	0	92	0.6848	1	0.42282
73.5	0	103.5	0.7101	1	0.44817
77.5	0	97.5	0.7949	2	0.43785
84	1	100	0.84	3	0.50299
71	0	94.5	0.7513	2	0.44654
84.5	1	114.5	0.738	2	0.52813
83	1	110	0.7545	2	0.52532
62.5	0	91.5	0.6831	1	0.39063
64.5	0	81.5	0.7914	2	0.43289
61.5	0	86.5	0.711	1	0.39172
78.8	0	102.8	0.7665	2	0.48944
72	0	98	0.7347	2	0.40223
81	1	99	0.8182	3	0.5625
65.5	0	98	0.6684	1	0.41456

67	0	91.5	0.7322	2	0.41615
64	0	88.5	0.7232	1	0.40506
74	0	97	0.7629	2	0.4625
83.5	1	107.5	0.7767	2	0.49118
74	0	97	0.7629	2	0.45122
78	0	107	0.729	1	0.46429
65	0	94	0.6915	1	0.38462
73.5	0	97.5	0.7538	2	0.45652
66	0	93	0.7097	1	0.40244
74	0	95	0.7789	2	0.45963
65	0	89	0.7303	2	0.40625
86.5	1	110	0.7864	2	0.51796
93	1	115.5	0.8052	3	0.52542
74	0	97	0.7629	2	0.44848
62	0	87.5	0.7086	1	0.4
69	0	97	0.7113	1	0.42331
72	0	93	0.7742	2	0.4472
95.5	1	107	0.8925	3	0.5753
82	0	97	0.8454	3	0.50932
84	1	106	0.7925	2	0.5122
67	0	89.5	0.7486	2	0.39181
55	0	83	0.6627	1	0.35484
88.5	1	118	0.75	2	0.5463
71.5	1	102	0.701	1	0.44136
65.5	0	92.5	0.7081	1	0.40184
95.5	1	123.5	0.7733	2	0.56176

Waist-height.Risk	Body.fat	Excess.body.fat	Glucose	Hyperglycemia	Total.cholesterol
0	28.7	0	86.35	0	131.7
0	37.7	1	96.02	0	157.63
0	21.8	0	87.46	0	169.87
0	28.6	0	87.26	0	154.26
0	27.7	0	86.67	0	122.02
0	28.9	0	92.29	0	158.87
0	13.1	0	93.51	0	105.72
0	27.9	0	87.87	0	182.6
0	31.2	0	84.5	0	128.76
0	35.2	1	89.72	0	111.62
0	32.4	0	93.77	0	123.9
0	36.7	1	91.33	0	153.93
0	22.4	0	85.67	0	142.3
0	38.2	1	90.38	0	161.48
0	36.4	1	86.54	0	140.89
1	46	1	91.63	0	182.05
0	6.1	0	82.13	0	106.45
1	31.3	1	90.19	0	159.41
1	36.1	1	81.21	0	138.12
0	19.2	0	91.2	0	193.23
0	8.4	0	88.78	0	161.41
0	11.6	0	86.58	0	130
0	15.19	0	86.16	0	177.08
0	16.4	0	91.54	0	162.09
0	13.8	0	80.03	0	193.85
0	15.9	0	75.84	0	95
0	14.5	0	92.44	0	138.14
0	7.4	0	89.93	0	178.65
1	41.9	1	90.43	0	140.87
0	27.6	0	89.7	0	182.39
0	32.3	0	93.12	0	144.25
0	23	0	85.25	0	164.01
0	19.6	0	87.04	0	154.88
0	30.8	0	92.82	0	155.12
0	11.5	0	85.77	0	179.04
1	31.6	0	89.18	0	152.09
0	23.4	0	93.92	0	136.66
1	41.3	1	109.4	1	142.21
1	37.4	1	96.11	0	155.82
0	16.9	0	83.18	0	150.06
0	12.3	0	85.29	0	208.5
0	10.2	0	88.75	0	145.08
0	30.3	0	84.02	0	120.33
0	30.2	0	93.98	0	162.56
1	27.8	0	94.73	0	183.2
0	27.3	0	90.11	0	271.33

0	22	0	90.04	0	164.38
0	15.7	0	80.98	0	142.63
0	28.5	0	89.96	0	160.4
0	36.1	1	83.14	0	121.72
0	17.1	0	88.23	0	143.34
0	36.4	1	100.14	1	162.06
0	26	0	106.36	1	166.28
0	26.1	0	102.54	1	161.69
0	22.3	0	84.41	0	195.77
0	25.9	0	94.73	0	177.69
0	19.4	0	95.84	0	167.45
1	42.3	1	97.9	0	154.1
1	31.8	1	97.92	0	182.56
0	32	0	89.19	0	174.54
0	18.3	0	84.27	0	168.91
0	27.1	0	92.73	0	157.2
0	27.7	0	81.66	0	143.41
1	24.5	1	94.71	0	155
1	27.6	1	92.2	0	163.16
1	35.4	1	87.5	0	158.04
0	24	0	100.81	1	141.63
0	8.9	0	85.22	0	139.46
1	43.3	1	90.43	0	213.47
0	29.2	0	89.32	0	154.23
0	21.7	0	92.56	0	148.56
1	43.9	1	95.17	0	117

Hypercholesterolemia	HDL-c	hypoalphalipoproteinemia	LDL-c	High.LDL-c	VLDL-c	High.VLDL-c
0	66.78	0	57.24	0	7.68	0
0	47.12	1	99.03	0	11.48	0
0	65.11	0	97.72	0	7.04	0
0	67.1	0	76.30	0	10.86	0
0	53.9	0	59.46	0	8.66	0
0	71.65	0	72.76	0	14.46	0
0	48.46	0	49.81	0	7.45	0
0	60.71	0	110.27	0	11.62	0
0	45.01	1	63.02	0	20.73	0
0	49.99	1	54.92	0	6.71	0
0	55.52	0	59.14	0	9.24	0
0	60.02	0	85.74	0	8.17	0
0	73.74	0	56.56	0	12.00	0
0	66.28	0	79.20	0	16.00	0
0	48.69	1	78.67	0	13.53	0
0	30.78	1	115.44	0	35.83	1
0	47.39	0	48.59	0	10.47	0
0	47.71	0	98.41	0	13.29	0
0	38.97	1	68.82	0	30.33	1
0	62.51	0	122.52	0	8.20	0
0	51.29	0	98.04	0	12.08	0
0	41.68	0	72.36	0	15.96	0
0	45.97	1	93.56	0	37.55	1
0	54.32	0	91.47	0	16.30	0
0	36.28	1	130.16	1	27.41	0
0	35.69	1	42.15	0	17.16	0
0	45.95	0	80.58	0	11.61	0
0	75.76	0	89.61	0	13.28	0
0	46.7	1	75.40	0	18.77	0
0	79.99	0	80.06	0	22.34	0
0	49.67	1	83.27	0	11.31	0
0	54.51	0	96.48	0	13.02	0
0	63.1	0	85.58	0	6.20	0
0	53.77	0	85.98	0	15.37	0
0	65.01	0	96.11	0	17.92	0
0	48.47	1	91.03	0	12.59	0
0	40.49	1	78.29	0	17.88	0
0	45.55	1	82.94	0	13.72	0
0	49.07	1	93.92	0	12.83	0
0	57.44	0	79.71	0	12.91	0
1	86.84	0	106.64	0	15.02	0
0	56	0	78.31	0	10.77	0
0	51.11	0	51.76	0	17.46	0
0	55.3	0	92.81	0	14.45	0
0	68.86	0	101.15	0	13.19	0
1	101.24	0	154.92	1	15.17	0

0	74.94	0	81.55	0	7.89	0
0	59.82	0	75.85	0	6.96	0
0	49.45	1	92.15	0	18.80	0
0	37.66	1	74.39	0	9.67	0
0	45.15	0	86.74	0	11.45	0
0	60.24	0	90.03	0	11.79	0
0	69.74	0	84.91	0	11.63	0
0	66.76	0	82.06	0	12.87	0
0	67.31	0	99.92	0	28.54	0
0	67.41	0	96.03	0	14.25	0
0	59.94	0	88.09	0	19.42	0
0	47.91	1	86.46	0	19.73	0
0	36.24	1	124.50	0	21.82	0
0	71.85	0	86.57	0	16.12	0
0	45.71	1	89.73	0	33.47	1
0	62.08	0	82.41	0	12.71	0
0	57.52	0	74.56	0	11.33	0
0	45.09	0	97.68	0	12.23	0
0	41.47	0	100.24	0	21.45	0
0	48.93	1	92.70	0	16.41	0
0	74.15	0	48.46	0	19.02	0
0	50.97	0	79.72	0	8.77	0
1	43.69	1	151.53	1	18.25	0
0	55.11	0	87.42	0	11.70	0
0	75.6	0	65.02	0	7.94	0
0	31.18	1	70.57	0	15.25	0

TGL	Hypertrygliceridemia	Insulin	HOMA-IR	Insulin.resistance	Adiponectin
38.42	0	8.01	1.708	0	10.864
57.39	0	7.06	1.674	0	4.56
35.18	0	7.99	1.725	0	9.099
54.31	0	14.28	3.077	1	5.305
43.3	0	8.03	1.718	0	4.845
72.28	0	12.42	2.83	0	7.773
37.24	0	8.69	2.006	0	2.542
58.08	0	7.67	1.664	0	10.6
103.63	0	16.04	3.347	1	2.523
33.54	0	8.27	1.832	0	6.221
46.2	0	5.87	1.359	0	3.679
40.86	0	12.45	2.808	0	2.736
59.98	0	17.64	3.731	1	6.604
79.98	0	11.37	2.537	0	4.486
67.65	0	11.1	2.372	0	3.079
179.16	1	30.29	6.853	1	4.264
52.34	0	11.14	2.259	0	2.933
66.44	0	7.14	1.59	0	2.303
151.67	1	11.82	2.37	0	1.743
41	0	7.89	1.777	0	3.669
60.41	0	9.18	2.012	0	3.934
79.81	0	13.29	2.841	1	1.956
187.76	1	7.29	1.551	0	2.375
81.5	0	11.1	2.509	1	4.282
137.07	0	9.18	1.814	0	2.647
85.81	0	38.45	7.2	1	1.121
58.07	0	9.52	2.173	0	2.368
66.38	0	8.27	1.836	0	2.874
93.85	0	19.53	4.361	1	2.671
111.72	0	10.05	2.226	0	2.819
56.57	0	11.33	2.605	0	2.131
65.09	0	6.38	1.343	0	5.481
31.01	0	9.55	2.052	0	4.403
76.84	0	14.77	3.385	1	3.151
89.62	0	6.42	1.36	0	2.118
62.94	0	12.61	2.777	0	1.952
89.4	0	13.14	3.047	1	3.081
68.61	0	14.92	4.03	1	3.861
64.13	0	20.4	4.841	1	2.063
64.57	0	9.74	2	0	3.701
75.11	0	7.63	1.607	0	4.732
53.86	0	9.1	1.994	0	4.346
87.28	0	8.84	1.834	0	10.559
72.25	0	11.1	2.576	1	3.43
65.97	0	11.14	2.606	1	3.185
75.83	0	14.81	3.295	1	8.772

39.46	0	10.65	2.368	0	3.491
34.81	0	5.55	1.11	0	3.516
93.99	0	17.45	3.876	1	2.549
48.35	0	9.33	1.915	0	3.194
57.24	0	8.12	1.769	0	1.848
58.97	0	9.52	2.354	0	3.267
58.16	0	12.13	3.186	1	5.966
64.35	0	8.43	2.134	0	2.612
142.7	0	17.35	3.616	1	8.982
71.24	0	13.26	3.102	1	2.368
97.1	0	16.83	3.983	1	3.761
98.66	0	34.32	8.296	1	2.416
109.09	0	18.97	4.587	1	1.718
80.62	0	12.51	2.755	0	5.143
167.37	1	11.45	2.382	0	1.067
63.54	0	9.04	2.07	0	4.128
56.63	0	9.93	2.002	0	4.233
61.15	0	19.87	4.647	1	2.022
107.27	0	8.42	1.917	0	3.298
82.05	0	24.82	5.362	1	1.573
95.08	0	12.65	3.149	1	2.236
43.84	0	8.53			2.84
91.23	0	22.4	5.002	1	1.803
58.48	0	10.91	2.406	0	4.356
39.71	0	9.97	2.279	0	3.989
76.23	0	67.2	15.791	1	1.427

Hypoadiponectinemia	IL-6	Kilocalories	Proteins(g)	Lipids(g)	SFA(g)	MFA(g)	PFA(g)
0	19.44	1583	59.13	53.2	13.7	18.5	8.43
0	0.84	2304.33	117.56	102.55	25.76	33.96	19.63
0	0.25	1154.33	44.73	44.19	15.7	14.9	8.83
0	0	1879.67	68.1	90.28	16.06	19.76	7.33
0	0	1528.33	69.87	66.17	20.77	24.33	8.57
0	0.51	1673.67	49.33	73.12	20.67	20.73	6.6
1	0.23	2811.33	219.96	78.38	19.66	25.56	10.56
0	0.67	2279.33	69.66	84.36	27.76	29.46	8.23
1	0	2916.33	108.16	87.88	26.36	26.9	11.03
0	0.19	2798	104.4	81.59	30.93	21.46	8.8
0	0.14	1687.33	101.9	65.61	18.46	19.96	6.4
1	0.19	1902.33	78.76	78.34	31.1	26.26	8.13
0	0.15	1764	95.6	43.95	7.4	9.9	20.1
0	2.46	2263.66	96.17	97.77	24.13	23.57	10.9
0	0	1296.67	57.8	32.22	11.27	10.9	4.53
0	1.9	1275.33	76.8	43.33	14.27	11.93	3.97
1	1.79	1508	73.9	60	14	21.8	8.3
1	1.73	1451.7	55.1	59.4	18.3	11.9	3.4
1	0	1784.3	65.8	68.7	28	18	4.4
0	0	2044	96.8	85.8	29	30.7	9.7
0	0	3346.3	103.4	163.5	27.8	13	14.2
1	1.74	2871.7	94	129.1	27.5	22.5	7.1
1	0	1586.3	71.7	72	20.4	30.2	11.5
0	2.08	2763.3	67.8	119.1	32.5	28.3	13.1
1	0	1962.7	84.6	70.4	17.2	26.9	8.5
1	0.16	2346	96.1	109.3	17.3	38.7	15.3
1	2.54	1331.3	60.8	45.4	12	15.6	5.6
1	0	1315	55.1	53.5	15.4	17.1	4.7
1	0	3199	69.2	86.4	12	16	9.4
1	10.98	2225	100.4	81.1	23.4	22.7	8
1	1.96	1042.7	35	34	6.7	13	6.5
0	2.34	2867.3	88.3	128.73	31.7	18.96	6.36
0	2.74	1838	54.53	87.4	21.63	13.66	3.5
0	1.71	2197.666	84.1	120.866	26.1	39.833	11.833
1	1.82	4028	150.9	134.52	50	75.6	14.1
1	2.03	2035.333	88	92.733	21.633	27.933	10.6
0	2.17	1711.333	79.3	60.1	16.566	13.933	12.4
0	2.25	1356.333	45.866	36.84	6.966	12.133	6.033
1	4.04	1207	58.066	41.85	8.766	14.666	4.1
0	2.97	1727.333	94.6	74.44	23.4666	25.733	7.233
0	2.94	1835.666	84.4	91.9	23.133	38.333	11.4
0	2.29	2363	93.433	84.246	20.866	18.666	3.966
0	2.06	1473	60.566	55.046	17.466	12	3.9
0	2.19	1479.333	52.066	38.566	7.533	16.533	8.6
0	1.82	2057.666	89.866	82.526	18.9	33.7666	15.3
0	3.89	1353.333	65.5	67.336	25.1	19.466	5.5

0	3.03	1816.66	107.1	52.313	21.033	20.0333	5.533
0	2.23	1300.333	67.466	48.47	11.8	13	6.033
1	2.34	1382	57.733	56.58	17	19.5	7.133
0	2.48	1432.66	54.266	52.823	13.433	19.266	9.2
1	2.82	2530	62	104.07	24.5	22.1	24.1
0	3.78	789.333	34.933	31.393	8.933	13.1	6.1
0	4.11	1389.6	62.033	63.843	19.366	18.9	7.96
1	3.39	1370.66	53.63	50.01	18.2666	13.73	4.53
0	2.42	1453.33	64.8	43.58	14.76	13.36	6.43
1	3.71	1426.3	70.1	40.38	8.46	13.36	5.766
0	3.05	2177.66	81.33	95.97	20.93	38.33	15.16
1	15.26	4939.66	278.56	283.56	89.13	107.03	47.5
1	4.97	1326.33	47.03	45.81	19.03	13.06	4.9
0	1.38	2700.33	88.46	127.02	34.13	43.16	18.83
1	4.24	1877	75.16	75.11	22.16	22.76	10.9
0	3.94	1165.66	62.46	44.42	17.33	14.83	5.5
0	1.18	1578	72.3	73.97	13.7	16.6	4.2
1	8.89	2903	105.8	126.08	33	43.3	16.13
0	4.97	1639	75.5	52.75	7.9	15.1	8.6
1	1.49	1777	76.23	80.9	23.63	25.16	8.96
1	1.61	2031.66	73	79.49	18.43	22.2	8.3
1	2.08	2063	104.9	69.04	26.15	20.55	7.1
1	2.51	1614.33	79.63	46.37	13.8	11.86	6
0	2.97	1574	77.87	46.19	14.23	12.1	6.1
0	3.55	1963.33	95.9	114.93	27.66	37	10.8
1	1.91	1993.33	72.83	66.55	19.3	23.63	9.03

Carboydrates(g)	Sugars(g)	Fiber(g)	Cholesterol(mg)	Vitamine.A	Vitamine.B1	Vitamine.B2
237.23	43.7	30.8	123	2319.67	1.37	1.47
255.36	32.5	4.33	480	765	1.53	1.59
147.17	14.47	11.27	321	1177.67	1.18	1.5
209.8	31.9	12.3	315.66	2011.66	1.08	1.316
175.53	26.57	17.9	295.67	710.33	1.19	1.39
210.5	27.7	10.53	156.33	889.67	0.68	0.87
306.26	16.76	30.43	524.66	3186.33	2.01	3.02
337.43	12.16	12.93	125	606	1.753	1.71
430.66	82.63	43.86	302.66	846	1.93	2.48
450.06	11.86	56.76	245.33	1152	3.41	3.1
170.66	40.16	25.13	563.33	2398.66	1	1.41
222.76	39.56	17.2	464.66	943	1.04	1.49
286.1	84.4	50.9	8	191	3.31	1.36
666.3	51.3	11.8	346.67	3835	1.32	1.4
203.83	51.8	16.17	181.33	401.67	0.86	1.26
145.97	34.2	25	153.33	835	0.94	1.06
173.3	44.9	11	199	635	1.1	1.5
180.4	12.5	8.7	117.3	295	0.8	1.1
240.5	37.1	7	373	457.7	2.1	2.6
235.9	32.7	17.9	473.7	474.7	1.6	2.2
379.4	24.9	15.2	109.3	91.7	2.7	3.2
363	62	12.1	234.7	1542.3	1.9	2.6
168.3	18.7	8.7	559.7	303.7	0.9	1.4
366.3	44.1	13.7	210.7	221.3	1	1.3
265.4	9.8	21	400	1023.7	1.4	1.1
278.3	27.8	8.7	280.3	251.7	1.1	2.5
182.8	16.6	11.1	134.3	175.3	1.1	1.1
165.8	12.3	10.8	125.3	421	0.9	1.2
302.3	68.4	21.1	198.7	2755	1.8	2
274.6	40	8.2	434.7	413	2	3.2
158	27.8	17	48.3	3582.7	0.9	0.6
331.86	26.2	10.56	556.66	458	0.77	1.08
211	24.93	7	133.66	998.33	1.27	1.12
205.166	26.2	15.566	332.333	525	1.586	1.28
572	16.6	14.8	395	1111	4.63	6.21
223.333	62.2	22.566	350.666	452.333	0.84	1.036
255.866	16.7	44.066	203.333	574.333	1.8	1.073
227.3	45.5	29	120	3149.333	1.4	0.996
156.933	48.566	9	279	753	0.436	0.456
169.966	24.566	15.533	298	631.333	1.52	1.316
179.933	28.666	11	286.3333	284.333	1.373	1.113
297.433	11.033	6.633	176.333	301.333	0.843	1.623
188.566	23.566	17.7	148	557.333	1.076	1.123
237.4	45.366	25.66	134	481	1.153	1.036
288.3	14.733	40.933	279	1676.666	2.523	1.333
129.333	24.3	8.666	195	363.333	0.903	0.99

244.066	13.966	17.4	217.666	127	1.21	1.69
148.3	11.133	19.133	151	341	0.846	1.033
164.4	30.166	12.5	314.333	469.666	1.06	1.333
189.066	24.1	12.433	229.666	982.666	1.393	1.306
346.1	94.3	25.8	76	33	1.24	0.59
86.4666	7.666	6.933	84.333	96	0.43	0.443
139.73	25.56	6.9666	272	799.666	0.9433	1.09
177.5	7.2	7.933	284.66	428.666	1.21	1.53
206.76	9.333	10.566	379.333	319	1.18	1.98
208.36	26.933	19.3	118.6	1100.66	1.046	1.01
249.56	31.86	20.5	327	701	1.45	1.53
308	36.13	20.36	6497.66	4422	2.55	10.06
197.23	32.83	16.36	161.66	598	0.72	1.2
306.96	65.6	15.1	320	754.3	1.36	1.5
226.4	30.53	11.16	206.33	545.66	0.97	1.383
129.63	17.83	4.16	519.33	1483	0.9	1.55
180.9	52.1	8.3	498	597	0.31	0.61
348.4	35.33	38.06	302	1068.66	2.17	1.33
214.9	18.8	11.3	125	922	0.65	0.52
194.7	18.766	10.66	371	580.66	1.16	1.6
270.23	47.43	19.93	159.33	2193	1.33	1.38
262.3	26.15	9.45	342.5	1241	1.27	2.3
225.3	18.6	25.5	211.33	1426.66	1.21	1.58
218.6	23.23	25.4	204	846	1.2	1.47
139.9	4.56	11.13	351.33	707.66	0.78	0.82
270.5	47.53	15.56	182	839	1.95	2.11

Vitamine.B6	Vitamine.B12	Vitamine.C	Vitamine.B9	Vitamine.B5	Vitamine.B3	Vitamine.E
1.81	1.88	574.5	266.77	2.28	10.38	5.83
1.51	2.84	62.96	413.9	2.67	15.53	3.43
1.36	2.29	45.87	255.7	4.313	43.295	1.17
1.156	3.083	31.466	227.46	1.13	11.25	1.42
4.31	3.16	94.1	194.63	2.87	12.81	4.43
0.56	1.92	24.87	162.6	2	6.15	0.66
4.33	25.18	237.03	534.76	7.75	66.19	5.48
1.2	1.81	57.8	253.63	1.72	15.18	2.02
2.46	27.35	308.4	514.33	2.58	31.67	4.8
3.13	5.24	1134.56	898.3	2.56	29.97	5.85
1.61	6.24	105.43	203.76	12.29	19.53	1.8
1.17	2.62	32.5	99.76	2.9	11.51	2.77
2	0.15	160.8	958.8	2.57	9.36	2.23
1.54	4.04	15.67	336.87	3.44	22.24	2.05
1.66	2.78	54.27	141.73	2.51	4.33	2.59
1.35	1.8	71.63	180.2	1.93	18.05	1.44
1.9	3.5	71.6	273.8	1.9	21.5	1.2
0.7	2.3	35.7	70.7	1.4	9.5	0.5
2.1	3.5	42.5	353.9	3.8	23.7	1.1
1.8	6.8	33.4	242.4	2.6	20.1	0.5
3.7	0.7	158.2	684.5	1.6	35.5	0.5
2.5	7.7	181.1	315.3	1.6	28.2	3
1.3	5.1	15.8	112.4	2.5	10	1.3
0.9	1.7	72.8	127.8	1.6	12.1	0.6
1.1	3.4	79.4	291.5	2.2	13.9	3.4
1.1	2.4	38	95.3	2.9	16.1	1.2
0.9	2.3	24	113.4	1.8	11.9	0.6
1	3.1	52.8	108.9	2	10.8	1.1
1.8	1.8	120.5	462.7	4.4	18.1	0.6
2	5.8	53.9	329.2	3	21.1	1
1	1.2	217.7	136.7	1.6	13.7	4.4
0.95	3.75	140	113.03	8.33	10.57	1.49
1.19	1.25	55.13	179	0.89	12.68	1.33
1.09	2.496	12.166	177.73	2.086	17.206	3.47
4.56	9.86	129.5	902.2	8.51	51.53	1.11
1.17	3.52	144.066	129.8	2.226	8.003	2.986
1.26	1.08	111.1	441.8	3.846	15.21	1.246
2.153	1.633	451.56	226.133	1.423	11.796	7.436
1.186	2.056	72	86.366	1.84	15.026	1.313
1.533	6.54	83.533	228.1	1.56	22.656	2.466
1.193	3.53	56.7	116.33	11.903	16.463	0.576
0.82	1.47	11.466	86.9	1.57	16.756	2.25
0.693	1.22	39.766	277.9	1.023	11.28	1.013
0.926	0.696	111.566	255	1.673	10.446	4.306
1.116	1.976	58.833	342.8	3.356	16.09	4.496
0.796	1.793	16.533	93.933	0.673	14.486	0.36

2.16	2.126	21.9	200.63	3.653	31.726	2.133
0.63	2.19	37.1	302.633	1.893	9.45	1.556
1.216	1.553	87.066	206.133	1.353	16.25	0.913
1.33	1.586	67.866	225.966	2.666	17.553	2.823
1.03	0.11	178.9	222.7	0.61	20.07	0.22
0.723	2.216	60.5	67.833	0.636	11.096	0.32
0.8	8.836	107.133	121.766	2.406	13.42	1.82
1.443	2.69	64.433	167.73	2.6633	13.833	2.13
1.41	5.223	57.733	242.16	1.57	15.28	1.64
1.38	1.72	91.7666	359.133	2.153	18.866	2.126
0.93	3.29	80.56	232.4	2.44	14.4	0.81
3.5	17.62	37.26	840.2	21.22	17.53	5.32
1.06	3.02	175.1	196.86	2.23	6.98	2.64
1.54	4.68	153.73	307.7	2.55	16.99	0.9
0.99	2.52	60.36	185.46	3.56	13.57	2.39
1.03	7.6	29.36	169.33	3.1	12.3	0.88
0.66	3.4	43.8	65.8	1.49	8.43	2.94
1.25	2.256	82.43	254.53	1.57	15.75	3.27
1.18	3.88	38.5	51.1	1.35	23.99	0.27
0.99	2.283	20.93	127.5	2.75	9.71	0.52
1.503	3.35	138.1	304.2	1.88	19.17	0.82
2.57	3.575	98.7	245.15	4.585	31.37	0.73
1.176	7.66	184.6	266.4	2.8	14.93	2.56
1.2	3.05	221.83	243.63	2.71	16.53	2.19
1.07	3.1	56.5	110.9	1.48	18.763	1.38
2.33	5.23	53.9	357.366	1.783	29.34	5.03

Ca	Fe	K	Mg	Na	P	Se	Zn	Sugar.equivalent.
874	13.19	3615.66	427	1587.67	658.33	32.67	5.37	2.87
1006.36	21.93	2906	367.66	1549	741.33	68.33	8.23	0.66
657.33	11.35	1973	181	1320	647.33	53.67	5.2	2.51
794.66	19.41	1125.66	199.66	2788	462	35.33	6.56	0
871	11.9	2390	274.33	1716.67	790.67	33	6.7	2.49
641.33	11.29	1264	297	2267.67	637	22	4.53	4.48
1867	26.68	6831.33	497	3351.666	1770.33	58.33	19.16	0
1163	15.35	2649.33	315.66	2771.33	757	54.33	6.3	12.753
1431.33	23.24	3703.33	445	3367.33	1420.33	111	14.16	3.91
2003.66	30.24	5755.33	487	2860.33	953.33	53.66	12.4	0.493
676.66	12.35	2602.33	258	2550.33	968	46.66	10.06	2.38
1373.33	12.09	1969.33	367	1567.33	995	72.33	6.5	2.06
743	25.97	5099	787	216	619	29	12.4	4.18
905.33	28.77	1429	183	2118	577	51	14.6	3.76
654.33	10.26	1651	158.33	911.33	696.33	17.33	4.31	6.51
548.33	10.61	2121.67	201.67	1603	624	22	6.87	0.39
728.7	16.3	2000	176.7	2350.7	475	25.7	5.8	2.1
821.3	8.7	1128.7	219	1365	868.3	12	4.4	3.1
840.7	19.9	1802	164.3	3058	890.3	28.7	8.2	3.2
1196	16.2	2078.3	464	3004	1265.3	34	12.5	3.4
340.7	51.9	1726	111.7	6160	462.3	25	3.8	0.9
796	28.8	1885	213	4409	366	22	11.2	6.8
721.3	12.5	1591	158.7	1496.3	608.3	47	6.5	2.5
996.3	11.6	2126.3	133	3722.7	628.7	61.7	4.7	2.1
562.3	15.4	1937.3	272.7	1230	805.3	39.3	9.2	7.3
2509	10.7	3989	103.3	2412.7	1775.7	37.3	5.5	5.1
624	13.5	1218.3	324	2239.7	535.7	13	5.2	0.2
766.3	7.5	1662	267.7	1592.3	616	16.7	5.6	0.9
909.3	32.6	1726.7	190.3	1958.7	526.7	24.7	8.2	0.8
1854	18.8	1853	265.3	2240.7	927.3	34.7	11.8	2.9
321.7	7.5	1603.7	144.7	1125.3	389.7	9.3	4.2	3.1
1007.66	13.43	2101.66	149.33	3068	509	19	7.93	1.54
575	11.86	1239	250.33	3521	827.66	29.33	6.43	2.33
780.333	10.18	1585.33	502.33	2922	761	47	7.533	1.843
2403	76.29	3701	294	6863	1825	43	20.2	0
634	9.57	1951.67	154.333	2057	562	33.333	4.733	6.486
439	15.93	2414	491.666	1620.333	1292.33	30.666	7.333	1.833
601.666	13.07	3442	264.666	442	317.66	13.666	5.766	0
318.333	5.39	1517	174.66	1829	460.66	13	3.033	3.53
579.666	15.37	2023.33	287.333	1343.333	514	45.333	9.9	2.486
738.666	14.926	1621	203.666	2087.33	627.666	22.333	8.666	4.223
1126.333	11.773	1896.67	100.333	2943.666	545.333	9	4.3	15.48
782.666	17.093	1549	168.66	550.333	371	18	6.333	1.106
819.66	11.963	2005.67	221.666	2059	642	35.666	4.233	3.04
782.333	23.496	2649.67	614.333	2418.666	1244.666	45.333	9.166	1.08
900	10.216	1210.67	99.666	1485.333	264	19.666	6.633	0.91

1011	11.086	3155.33	256.333	3003	1017	12.666	6.033	12.3
679	10.743	1570.33	184.666	1796	672.666	20	6.033	0.443
851.666	16.673	1182.33	104.666	1545.333	435.666	27	3.933	2.71
801	13.31	1876.67	242.333	1654.666	472.666	41.333	4.633	2.756
302	8.25	2951	210	2137	447	5	6.8	7.58
182.666	5.543	1053.33	194.333	590.666	239.333	12.666	4.566	0
833.66	7.3766	1404	146.6666	1536.6666	636.66	22.66	3.433	0.36
675.33	13.27	1288	123	2060	482.66	32	5.66	1.85
694.33	25.59	1601.67	136.666	2446.6666	505.666	19.66	6.666	2.403
472.66	16.316	1823.66	248	1162.33	463.3333	24	6.26	3.89
926	14.49	1990.66	393.33	2413.66	883.66	41	6.23	4.05
2737.66	38.46	4963	443.66	7852.33	4058	444.33	26.23	3.13
824	8.07	2194	170.66	794	538	24	4.9	1.7
1038	16.66	2320.66	167.66	4414	761.33	29	9.9	5.97
1055.66	10.8	1934	170.66	2883	849.66	8.33	4.83	4.46
701.33	12.483	1344.6	163.3	1095.33	772.33	35	4.9	3.87
333	8.64	974	75	1226	390	43	9.2	4.75
1118.66	16.43	2914.6	329	3226.33	497.66	62.33	5.8	4.2
459	10.35	1356	124	2367	200	22	3.2	0
1242	12.71	1666	264	2520.33	922.33	23.66	4.2	2.91
846.66	19.65	2036.66	287	2151.66	720.33	13.33	4.63	3.06
1221	20.925	2778.5	364.5	4562	1389	38.5	9.8	9.92
1119.66	13.93	2627	324.33	12560.33	891.33	22.66	4.2	1.39
1100	13.98	2573.67	289	1684.67	928.67	25.33	4.7	1.93
372.33	12.74	1621.66	131.66	3746.66	388.66	16	6	0.55
983.66	20.07	1187.33	103.33	2182.33	580.66	24.33	11.06	4.24

Meat.equivalent	Cereal.equivalent	Fruit.equivalent	Fat.equivalent	Milk.equivalent
2.93	3.51	7.41	4.06	0.98
7.54	8	2.21	3.58	0.92
2.74	5.18	0.49	4.32	4.31
5.66	11.01	0.5	4.51	0.32
4.76	5.64	0.86	5.76	1.68
2.2	8.52	0.36	7.62	1.25
23.04	13.3	0.913	3.276	2.56
3.11	10.443	0.69	1.26	0.46
5.39	17.86	4.093	3.94	1.32
6.44	9.63	16.27	2.7	1.6
10.19	3.65	1.99	2.37	0.64
6.1	7.813	0.786	3.08	1.35
0.54	5.52	4.24	0	0.18
10.07	11.88	0	3.49	0
4.33	4.71	1.74	1.86	1.31
6.72	5.38	1.52	2.08	0.36
7.2	6.5	2	3.5	1
3.6	7.1	0.8	3.8	1.3
3.8	10.7	0.8	0.8	1.9
9.1	9.1	0.6	2.9	1.1
3.9	16.5	1.2	5.6	0.5
6.4	14.3	0	3.1	0.6
6.5	6	2.1	4.6	1
3.4	18.1	1.6	3.3	0.8
6.6	9.4	0.9	5.6	0
6	9	0	7.8	0
4.3	9.2	0.6	2.3	0.9
3.4	6	1.2	2	1
3.8	16	2.1	0.7	0.8
8.3	13.1	0.3	2.5	2.7
2	4.8	2.3	1.2	0
6.87	14.77	2.94	1.55	0.79
4	8.12	0.63	5.43	0.4
6.086	8.536	0.033	3.86	1.013
6.52	33	0	2.18	6
8.423	4.263	3.256	4.546	0.983
3.716	10.796	1.34	3.3	0.32
2.41	4.066	8.026	0.976	0.16
6.37	5.363	1.496	3.623	0.016
9.806	4.79	1.77	1.326	0.47
9.103	7.453	0.406	6.926	0.16
6.886	5.653	0.046	3.04	0.333
4.176	8.006	1.063	0.333	0.64
1.916	6.576	2.66	3.436	0.733
3.98	13.61	0.78	7.76	0
6.413	4.756	1.166	1.246	0.846

8.69	3.396	1.18	3.136	0.986
4.913	5.203	0.393	1.226	0.586
5.64	6.186	1.08	3.25	0.32
4.353	7.76	1	3.156	0.303
3.76	15.29	0.31	4.29	0
3.646	4.016	0.93	2.623	0
5.916	4.893	2.4	4.603	0.7
3.593	8.24	0.3466	1.936	1.253
5.22	9.55	0.643	2.846	0.96
5.34	6.203	0.7933	2.95	0.26
4.26	8.62	1.13	7.36	1.21
32.37	9.65	1.65	4.51	2.96
1.64	5.39	3.46	0.86	1.9
6.19	10.63	1.35	8.37	0.97
4.1	8.26	1.13	5.84	1.66
5.85	4.31	0.323	1.82	1.26
8.21	5.72	1.37	0.2	0
5.14	10.93	0.95	8.61	1.26
7.28	11.2	0	0.47	0
6.35	8.79	0.27	4.96	1.25
4.92	8.42	2.34	4.69	0.95
8.41	7.59	0.675	3.35	1.8
4.21	7.01	1.67	0.92	1.36
4.94	6.04	2.19	1.08	1.45
10.83	5.023	0.94	6.2	0.01
5.81	11.7	0	5.2	0.96

Legume.equivalent	vegetable.equivalent	Physical activity
0	3.99	0
2.36	43.06	0
0	4.33	1
0.35	1.24	0
0.72	2.63	0
0.39	1.95	0
0.29	7.38	1
0.49	0.49	0
1.21	3.84	0
0.65	2.506	1
1.26	4.473	1
0.39	2.32	1
8.44	2.86	0
0	2.83	0
0.77	1.48	0
0.89	1.96	0
0.3	1	0
0.1	2.1	1
0	0	0
0	3.4	0
0	0.3	0
0	2	0
0	0.3	1
0	0.4	0
1	2.3	1
0.1	1.9	0
0.3	0.8	1
0.1	2	1
0.2	0.7	0
0	0.1	1
0	3.6	1
0.07	1.23	0
0.1	1.46	0
0.606	0.98	1
0	0.72	1
0.763	2.34	0
2.526	0.706	0
0.616	6.99	1
0	1.8	1
0.726	3.623	0
0	2.136	1
0.306	0.376	0
1.41	1.3	1
1.633	2.096	1
1.403	4.34	1
0.273	0.103	1

1.633	0.576	1
1.59	0.886	0
0.34	0.68	1
0.22	2.663	0
0.7	3.41	1
0	0.876	0
0	0.6166	1
0	4.1066	1
0	4.183	1
1.613	3.733	0
1.58	1.21	0
1.046	1.04	1
0.7267	1.14	1
0.72	1.21	1
0.19	4.27	1
0	0.67	1
0	1.16	0
3.92	2.79	0
0	7.51	0
0	1.286	0
0.7	2.68	0
0	3.195	0
1.35	4.17	1
1.31	4.62	1
0.07	1.4	1
0.56	2.01	0
